# A novel *in vitro* system of supported planar endosomal membranes (SPEMs) reveals an enhancing role for cathepsin B in the final stage of Ebola virus fusion and entry

**DOI:** 10.1128/spectrum.01908-23

**Published:** 2023-09-20

**Authors:** Laura Odongo, Betelihem H. Habtegebrael, Volker Kiessling, Judith M. White, Lukas K. Tamm

**Affiliations:** 1 Center for Membrane and Cell Physiology, University of Virginia, Charlottesville, Virginia, USA; 2 Department of Molecular Physiology and Biological Physics, University of Virginia, Charlottesville, Virginia, USA; 3 Department of Cell Biology, University of Virginia, Charlottesville, Virginia, USA; University of Arizona, Tucson, Arizona, USA

**Keywords:** filovirus, Ebola virus, enveloped virus, non-enveloped virus, viral membrane fusion, genome entry, endosomes, endosomal receptors, hemorrhagic fever viruses, Lassa, influenza, acid activated toxins

## Abstract

Ebola virus (EBOV) causes a hemorrhagic fever with fatality rates up to 90%. The EBOV entry process is complex and incompletely understood. Following attachment to host cells, EBOV is trafficked to late endosomes/lysosomes where its glycoprotein (GP) is processed to a 19-kDa form, which binds to the EBOV intracellular receptor Niemann-Pick type C1. We previously showed that the cathepsin protease inhibitor, E-64d, blocks infection by pseudovirus particles bearing 19-kDa GP, suggesting that further cathepsin action is needed to trigger fusion. This, however, has not been demonstrated directly. Since 19-kDa Ebola GP fusion occurs in late endosomes, we devised a system in which enriched late endosomes are used to prepare supported planar endosomal membranes (SPEMs), and fusion of fluorescent (pseudo)virus particles is monitored by total internal reflection fluorescence microscopy. We validated the system by demonstrating the pH dependencies of influenza virus hemagglutinin (HA)-mediated and Lassa virus (LASV) GP-mediated fusion. Using SPEMs, we showed that fusion mediated by 19-kDa Ebola GP is dependent on low pH, enhanced by Ca^2+^, and augmented by the addition of cathepsins. Subsequently, we found that E-64d inhibits full fusion, but not lipid mixing, mediated by 19-kDa GP, which we corroborated with the reversible cathepsin inhibitor VBY-825. Hence, we provide both gain- and loss-of-function evidence that further cathepsin action enhances the fusion activity of 19-kDa Ebola GP. In addition to providing new insights into how Ebola GP mediates fusion, the approach we developed employing SPEMs can now be broadly used for studies of virus and toxin entry through endosomes.

**IMPORTANCE:**

Ebola virus is the causative agent of Ebola virus disease, which is severe and frequently lethal. EBOV gains entry into cells via late endosomes/lysosomes. The events immediately preceding fusion of the viral and endosomal membranes are incompletely understood. In this study, we report a novel *in vitro* system for studying virus fusion with endosomal membranes. We validated the system by demonstrating the low pH dependencies of influenza and Lassa virus fusion. Moreover, we show that further cathepsin B action enhances the fusion activity of the primed Ebola virus glycoprotein. Finally, this model endosomal membrane system should be useful in studying the mechanisms of bilayer breaching by other enveloped viruses, by non-enveloped viruses, and by acid-activated bacterial toxins.

## INTRODUCTION

Ebola virus (EBOV), a negative-sense, single-stranded RNA virus belonging to the *Ebolavirus* genus within the *Filoviridae* family, causes a hemorrhagic fever associated with fatality rates of up to 90% (1). EBOV infections continue to be a major threat to human health, particularly in parts of Central and West Africa. There are currently three approved vaccines against EBOV: Ervebo [Food and Drug Administration (FDA) approved] (2), Zabdeno/Mvabea (approved for medical use in the European Union) (3, 4), and Ad5-EBOV vaccine (approved by the China Food and Drug Administration for emergency use) (5), but no vaccine is approved for other filoviruses, including *Sudan ebolavirus*, which was responsible for the 2022 Ebola Outbreak in Uganda (6) nor for *Marburgvirus*, which recently caused infections in Equatorial Guinea and Tanzania (7, 8). During the 2018–2020 EBOV Outbreak in the Democratic Republic of Congo, four investigational agents were evaluated in the Pamoja Tulinde Maisha (“Together We Save Lives” in Kiswahili) study. Two products demonstrated efficacy against EBOV: Ebanga (also known as mAb114), which is a single monoclonal antibody, and Inmazeb (also known as REGN-EB), which comprises a cocktail of three monoclonal antibodies (9). The U.S. FDA approved both medications for Ebola virus disease therapy (10, 11). As EBOV has been reported to persist in immune-privileged sites (12, 13), a promising treatment strategy would be to combine monoclonal antibodies with small molecules to completely clear the virus (9). It is also worth noting that the currently available vaccines and treatments are injectables, and there remains a need for easily administered (e.g., oral) anti-filoviral drugs (14). Gaining a better understanding of the virus is essential to guide the development of additional antiviral therapies against EBOV and other filoviruses. One route to anti-viral therapies, which has proven successful in combatting other viral infections, would be to develop novel entry inhibitors. To do so, there is a need to better understand the mechanism of EBOV cell entry.

EBOV entry into host cells is mediated by the viral glycoprotein (GP), which resides on the virion surface. GP is a class I fusion protein comprising two subunits: a receptor-binding subunit (GP1) and a fusion subunit (GP2) (15). Following attachment to the cell surface, the virus particles undergo internalization by macropinocytosis and are trafficked to late endosomes/lysosomes (15, 16). In this compartment, GP1 is cleaved by the low-pH activated endosomal cysteine proteases cathepsin B and cathepsin L, removing the heavily glycosylated mucin-like domain and glycan cap to generate a cleaved form of GP1 (approximately 19 kDa) (17 – 19). The cleavage of GP1 exposes the receptor-binding site for the EBOV endosomal receptor, Niemann-Pick type C protein 1 (NPC1) (20 – 23). After binding to NPC1, GP2 goes on to facilitate fusion of the viral and endosomal membranes by means of a poorly understood mechanism (24 – 26), which appears to be more complex than that of most other enveloped viruses that fuse at low pH (27, 28).

Low pH and GP1 interaction with NPC1 are both necessary but apparently not sufficient to induce complete EBOV membrane fusion (22, 29 – 31). Recent work by Munro and coworkers (32) suggests that upon binding to NPC1, GP1 undergoes conformational changes that enable GP2 to mediate the lipid mixing stage of fusion (hemifusion), in a process that also requires low pH and is enhanced by Ca^2+^. Ca^2+^ has also been shown to have direct effects on the fusion loop found within GP2 (33, 34). However, the infectivity of EBOV particles containing cleaved GP1 remains sensitive to a general cysteine protease inhibitor (E-64d), as well as agents that raise endosomal pH (35, 36). This suggests that an additional step is required for full and efficient fusion leading to a fusion pore that can transmit the viral RNA into the cytoplasm to initiate replication. This additional step has not yet been identified, and it remains unclear what the additional trigger is for full fusion and if it can only be found in late endosomes/lysosomes. Addresssing these questions has proven difficult in large part because full fusion has only been monitored in intact cells using infectivity as a surrogate readout. The ability to measure the EBOV GP-mediated full fusion reaction (i.e., fusion of both leaflets of the bilayers) in an open cell-free system with accessible endosomal membranes would be beneficial because different factors could be added or omitted, and thus their effects on fusion *per se* could be individually investigated at the experimenter’s will. To date, such a system has not been developed.

To overcome this deficiency, we developed a novel target membrane system with which to study fusion of virus particles with NPC1-containing late endosomes displayed on a supported planar membrane, which we term a supported planar endosomal membrane (SPEM). We then used SPEMs in an *in vitro* fusion assay that employs total internal reflection fluorescence (TIRF) microscopy to visualize binding and fusion (both hemifusion and full fusion) between fluorescently labeled (pseudo)viruses bearing a viral fusion glycoprotein and SPEMs. We first validated the system with an influenza virus [fowl plague virus (FPV)] and with pseudoviruses containing GP from the arenavirus Lassa fever virus. After establishing and validating this new *in vitro* fusion assay with viruses with well-understood fusion behavior, we proceeded to show that full fusion mediated by 19-kDa EBOV GP is dependent on low pH and enhanced by Ca^2+^, consistent with and extending other studies (26, 33). We also found that SPEMs retain some membrane-associated cathepsin activity, that E-64d (an irreversible cathepsin inhibitor) inhibits EBOV GP-mediated full but not hemifusion, that the reversible cathepsin inhibitor VBY-825 reversibly inhibits fusion, and that the addition of cathepsin B enhances fusion.

## RESULTS

### SPEMs as fusion targets for endosome-entering viruses

**FIG 1:**
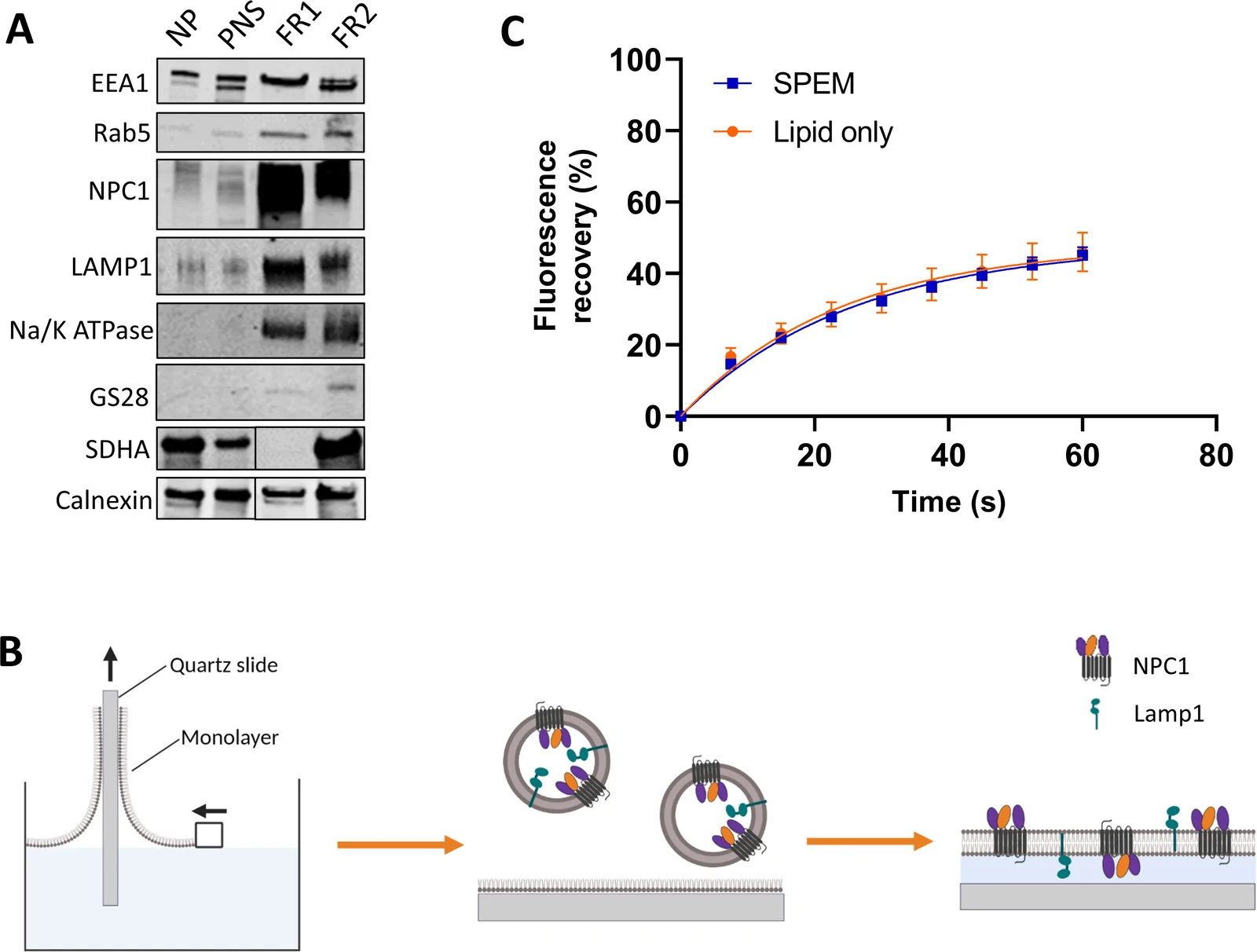
Preparation of SPEMs. (**A**) Immunoblots of gradient fractions probed for different organelle markers: early endosomes (EEA1 and Rab5), late endosomes, endolysosomes (NPC1 and Lamp1), plasma membrane (Na/K ATPase), Golgi apparatus (GS28), mitochondria (SDHA), and endoplasmic reticulum (ER) (calnexin). These are representative immunoblots from two independent endosome enrichment preparations. Preparation 1 was analyzed for EEA1, Rab5, NPC1, Lamp1, Na/K ATPase, and GS28. Preparation 2 was analyzed for calnexin and SDHA. For the latter, the NP/PNS and FR1/FR2 samples were separated by intervening lanes; hence, in the immunoblots of calnexin and SDHA, the NP/PNS and FR1/FR2 lanes were spliced, as indicated by the black vertical line. FR1, 7%–14% optiprep interface; FR2, 14%–25% optiprep interface. (**B**) Cartoon showing preparation of SPEMs. A lipid monolayer was transferred onto a clean quartz slide by immersing and pulling it through a monolayer of a lipid mix of brain PC, cholesterol, and DPS in the ratio 77:20:3. Next, the lipid monolayer covered slide was assembled into a watertight flow cell, and endolysosomes from FR1 were injected into the flow cell. The endolysosomes spontaneously spread on the supported monolayer to form SPEMs. (**C**) FRAP experiment on SPEMs. Carboxy-fluorescein-phosphatidylethanolamine (PE) was included in the monolayer prior to preparation of the bilayer. Total fluorescence intensities during patterned FRAP experiments from SPEMs as well as from lipid-only bilayers of brain PC and cholesterol (4:1). At least 10 regions on four independently prepared lipid-only bilayers and SPEMs were sampled to determine the average values reported. Fluorescence is scaled to the intensity range before and immediately after bleaching. DPS, 1,2-dimyristoyl-*sn*-gycero-3-phosphatidylethanolamine-PEG3400-triethoxysilane; FR1, fraction 1; FR2, fraction 2; FRAP, fluorescence recovery after photobleaching; NP, nuclear pellet; PC, phosphatidylcholine; PNS, post-nuclear supernatant; SDHA, succinate dehydrogenase complex subunit A.

We developed a new system employing endosomal membranes in a supported lipid bilayer format (SPEMs) as target membranes for measuring hemifusion and full fusion of viral particles that fuse in endosomes. This open *in vitro* system affords facile access to examine the roles of candidate fusion triggering factors that normally function within endosomes. To prepare SPEMs (see Methods for details), we homogenized HEK 293T cells, removed nuclei by centrifugation, and fractionated the resultant post-nuclear supernatant. The workflow to obtain an endosome enriched fraction is shown in Fig. S1. Analysis on a continuous OptiPrep gradient indicated that late endosomes are enriched in the range of 7%–14% OptiPrep (fractions 3–7 in Fig. S2). We, therefore, separated the post-nuclear supernatant on an OptiPrep 7%/14%/25% step gradient and consistently found that the 7%/14% interface is enriched for the late endosome markers NPC1 and Lamp1 [fraction 1 (FR1) in Fig. 1A]. This fraction also contains some early endosome, plasma membrane, and endoplasmic reticulum markers, which is consistent with previously published endosome enrichment schemes (37). The visible band at the 7%/14% Optiprep interface was collected, dialyzed against HMA pH 7.4 buffer, and used to prepare SPEMs.

To prepare SPEMs, a polymer-supported lipid monolayer was transferred to a quartz slide (38), and an aliquot of the endosome enriched sample was added to the monolayer to prepare the second leaflet of the supported membrane (Fig. 1B). This approach is similar to our previous approaches to display plasma membranes as target membranes for HIV fusion (39, 40) and reconstituted membranes to study SNARE-mediated exocytosis (41). Immunofluorescence analysis indicated that SPEMs contain both NPC1 and Lamp1 with many of their ectodomains facing outward, but also some in inward facing orientations (Fig. S3). Fluorescence recovery after photobleaching (FRAP) indicated that the SPEMs exhibit long-range lateral lipid mobility with a diffusion coefficient of 1.65 ± 0.1 × 10^−9^ cm^2^/s, consistent with lipid diffusion in a fluid lipid bilayer (Fig. 1C). The observation of lateral diffusion over many microns also means that SPEMs consist of lipid bilayers that extend over large areas on the planar support. Since a significant fraction of the late endosomal membrane proteins NPC1 and Lamp1 are both displayed in an outward facing orientation in the SPEMs, they are accessible for interaction with viruses approaching the SPEM surface.

**FIG 2:**
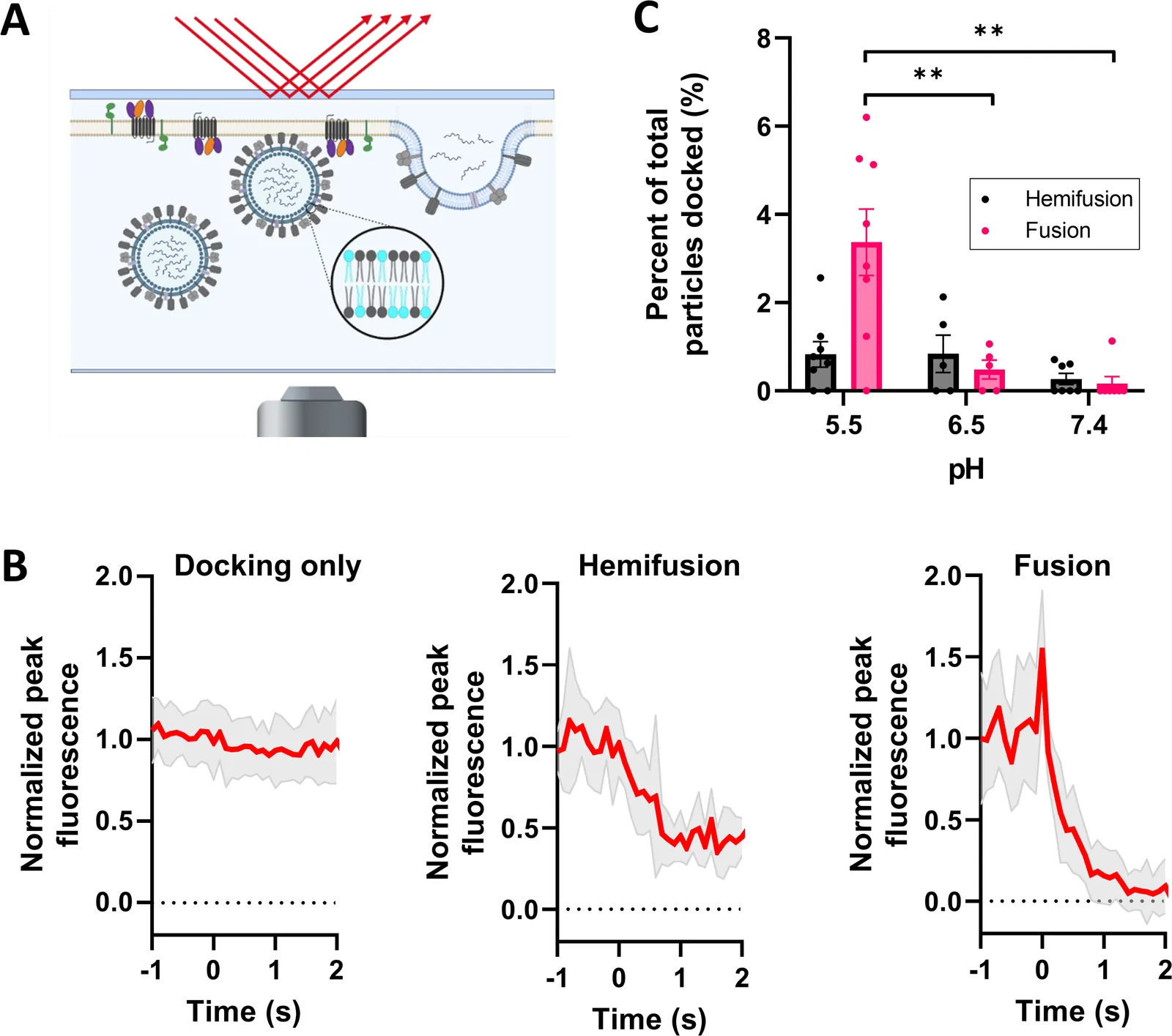
Influenza virus (FPV) fusion to SPEMs. (**A**) Cartoon depicting DiD-labeled FPV fusion to SPEMs. FPV was labeled with DiD during viral production; hence, the membrane label (cyan) is present in both leaflets. (**B**) Intensity traces of particles undergoing docking only (23 traces from pH 7.4 condition), hemifusion (7 intensity traces from pH 5.5 condition), and full fusion (21 intensity traces from pH 5.5 condition) were aligned and averaged (red traces). The shaded area represents standard deviation. (**C**) pH dependence of hemifusion (black) and full fusion (pink) of DiD-labeled FPV to SPEMs within a 3-min time period. Movies were recorded at a frame rate of 100 ms for a minimum of 2,000 frames. The total number of docked particles was quantitated for each condition. Each data point represents events observed on one separately prepared SPEM. Error bars indicate standard error. Statistical comparison was performed using Welch’s two-tailed *t* test. **P < 0.01. All comparisons not shown are not significant.

As a first proof of concept, we assessed the ability of fowl plague virus, an influenza virus, to fuse with SPEMs. To do this, we incorporated the membrane dye 1,1'-dioctadecyl-3,3,3',3'-tetramethylindodicarbocyanine (DiD) into the FPV membrane during virus production, added the particles to SPEMs, and monitored fusion events by TIRF microscopy for at least 2.5 min (Fig. 2A). Three types of events were observed: docking (binding) to the SPEM, hemifusion, and full fusion (Fig. 2B). Docking was characterized by the sudden appearance of punctate fluorescence, hemifusion by a decrease in fluorescence intensity to about half the initial intensity, and full fusion by the decrease in fluorescence intensity to background fluorescence. The distinction between hemifusion and full fusion is possible in this case because both leaflets of the particle envelope were labeled with DiD using the protocol of label incorporation during virus production (39). A partial loss of fluorescence might also be explained by transient fusion events (kiss and run events). However, this seems highly unlikely for the following reasons: the observed intensity peak in the full fusion events shows that most fluorophores are transferred from a spherical membrane to a planar membrane within 100 ms before they diffuse out of the region of interest (42). Opening and closing of a fusion pore would have to happen within this time and in a way that reproduces the observed narrow distribution of the fluorescence decay to around 50% of the starting intensity. As expected for influenza virus (43), fusion to SPEMs was highly pH dependent: an approximately 6–8 times higher fusion probability was observed at pH 5.5 when compared to pH 6.5 or 7.4 (Fig. 2C).

**FIG 3:**
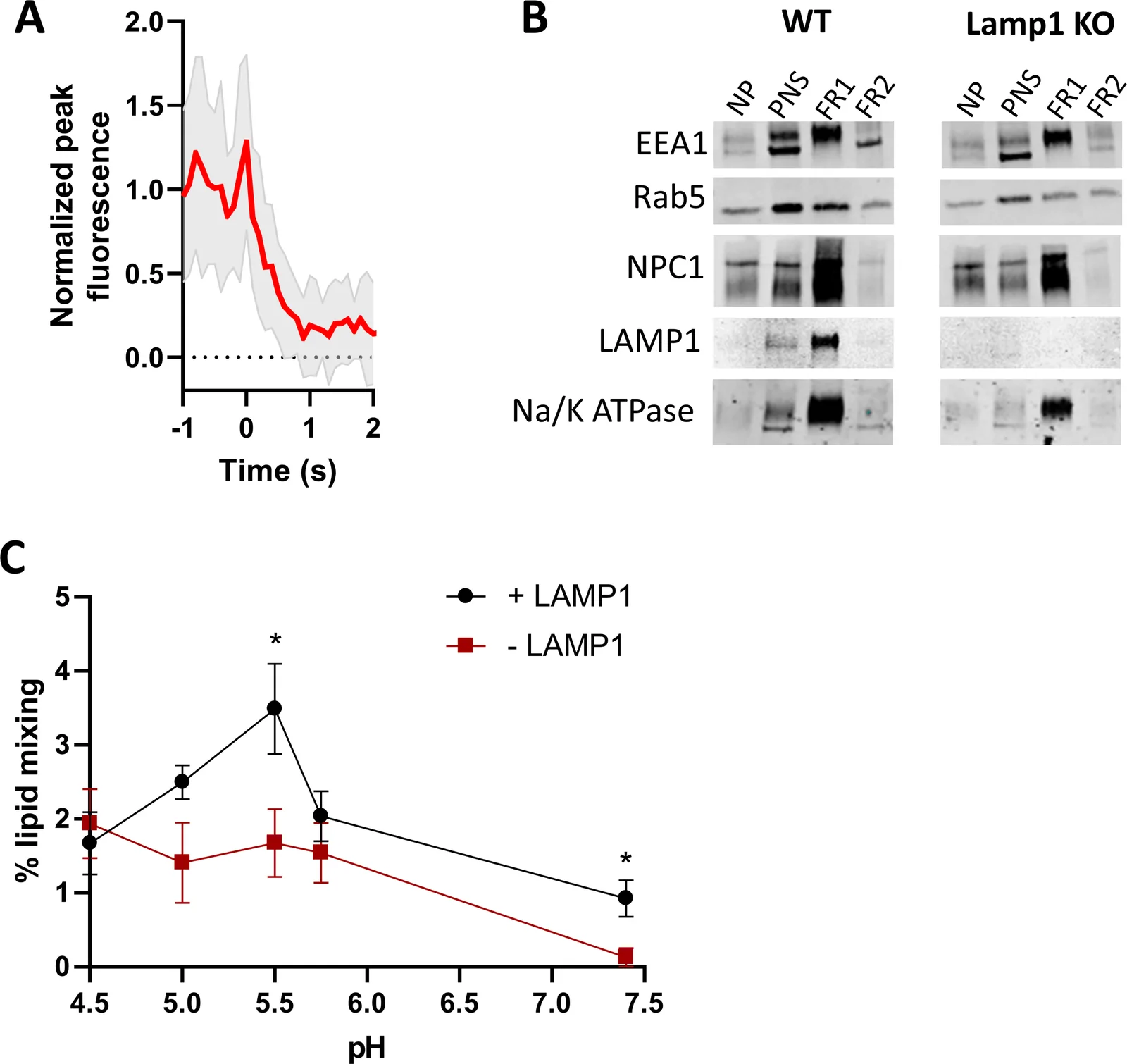
Lassa virus GP pseudovirus fusion to SPEMs. (**A**)Twenty intensity traces of fusion of HIV pseudovirions bearing Lassa GP, labeled with Atto488-DMPE in the outer membrane leaflet, were averaged. (Particles in this average curve were taken from the pH 5.5, no Lamp1 condition.) (**B**) Immunoblots of gradient fractions from HEK293T WT and Lamp1 KO cells probed for different organelle markers as denoted in the legend of Fig. 1. (**C**) Fraction of particles bearing Lassa GP that underwent lipid mixing with SPEMs with or without Lamp1. The virus particles were introduced into the flow cell at the indicated pH. Recording began immediately after input of viral particles into the flow cell. Data from five to nine SPEMs were averaged under each condition. Data from individual SPEMs are shown in Fig. S5C and D. Error bars indicate standard error. Welch’s two-tailed *t* test is shown above data. *P < 0.05. All comparisons not shown are not significant.

We next tested SPEMs as targets for fusion of pseudoviruses. For this, we labeled the membranes of HIV pseudovirus particles bearing the Lassa virus (LASV) glycoprotein with a fluorescent lipid, Atto-488-dimyristoylphosphatidylethanolamine (DMPE). As these particles were labeled post-pseudovirus production, the lipid dye is only found in the outer leaflet of their membrane envelopes (40). Thus, we only observed lipid mixing between the viral outer leaflet with the SPEMs in these experiments (Fig. 3A). Fluorescence decay time constants were determined for each event observed in the single particle recordings to differentiate between lipid mixing and undocking events (Fig. S4). We assessed the ability of the LASV GP pseudotyped particles to fuse with SPEMs at different values of pH. As expected, minimal fusion was seen at pH 7.4 but was clearly detected at pH 5.75 reaching a peak at pH 5.5 and then decreasing with SPEMs derived from wild-type (WT) cells (Fig. 3C; black data points). Since Lamp1 is the endosomal receptor for LASV (44), we examined fusion to SPEMs prepared from cells in which Lamp1 had been knocked out using CRISPR/Cas9 gene editing (Fig. 3B) (45). We previously showed that Lamp1 expressed on the plasma membrane enhances pseudovirus-to-cell and cell-to-cell fusion at pH 5.5 (45). As seen in Fig. 3C, at pH 5.5, more fusion is seen to SPEMs from WT cells than to SPEMs derived from Lamp1-negative cells. We also assessed binding of LASV GP pseudotyped particles to SPEMs with or without Lamp1 and observed modestly increased binding to both sets of SPEMs with reduction in pH (Fig. S5), suggesting that SPEMs contain additional attachment factors for LASV GP.

### Ebola virus glycoprotein-mediated membrane fusion with SPEMs

**Fig 4:**
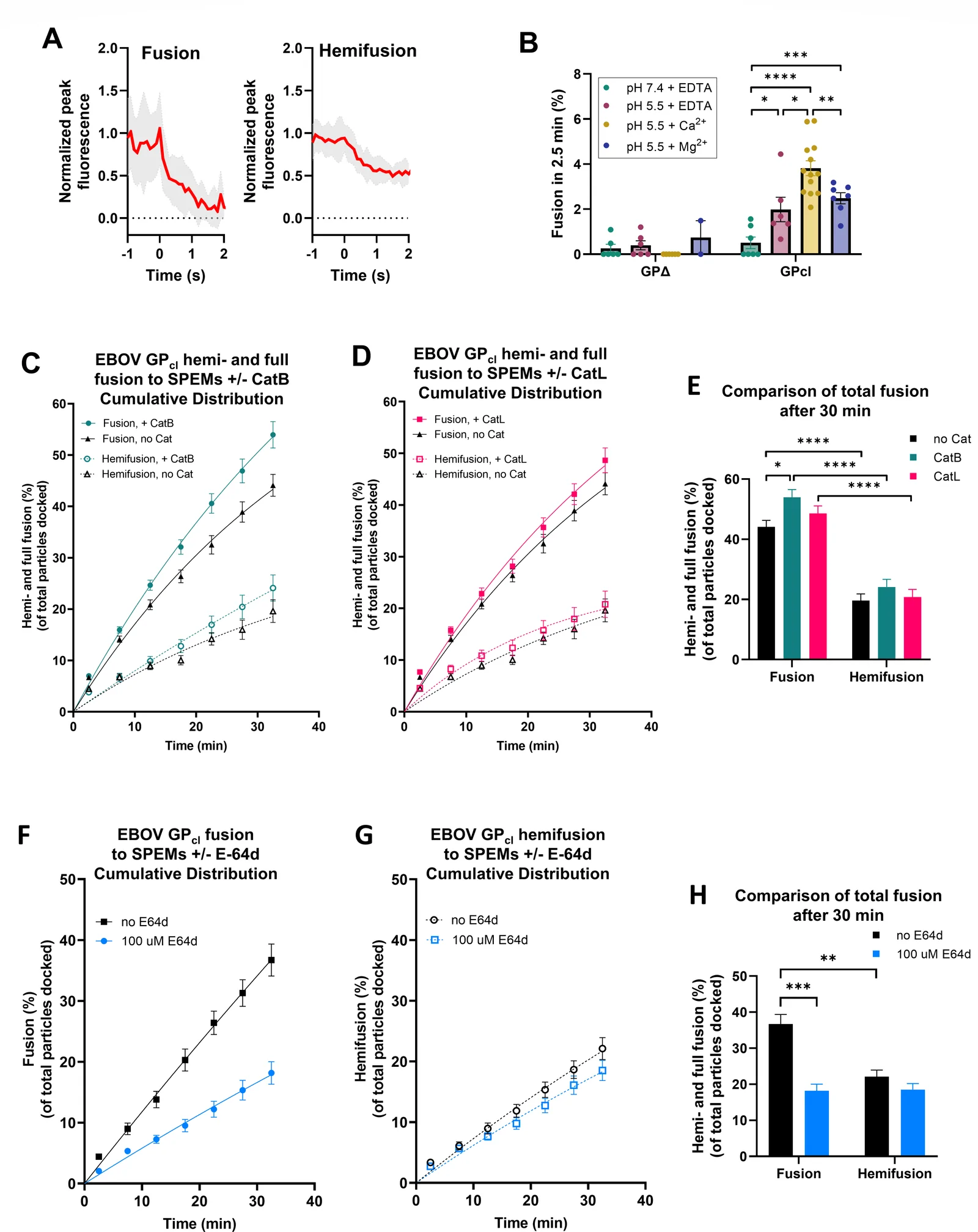
Ebola virus GP pseudovirus fusion to SPEMs. (**A**) Intensity traces of DiD-labeled EBOV GPcl pseudovirions undergoing hemifusion (23 intensity traces from pH 5.5, EDTA condition) and full fusion (21 intensity traces from pH 5.5, EDTA condition). Ebola pseudoviruses were labeled with 1-µM DiD during viral production so as to label both membrane leaflets. Traces were aligned and averaged (red traces). The shaded area represents standard deviation. (**B**) DiD-labeled HIV particles pseudotyped with Ebola GPΔ, treated with thermolysin to generate 19-kDa GP1 (GPcl) or untreated (GPΔ), fusing with SPEMs at pH 7.4 and pH 5.5 in the presence of 1-mM EDTA or pH 5.5 in the presence of Ca^2+^ or Mg^2+^ (0.5-mM CaCl_2_ or MgCl_2_). Each data point represents the average percent fusion of approximately 70–200 docked particles observed on each SPEM at 2.5 min post-adding low pH buffer. Statistical comparison was performed using Welch’s two-tailed *t* test. *P < 0.05, **P < 0.01, ***P < 0.001, ****P < 0.0001. All comparisons not shown are not statistically significant. (**C through E**) Cumulative distribution function for full fusion and hemifusion of EBOV GPcl pseudovirus with SPEMs in the presence or absence of added cathepsin B (0.4 µg/mL) (**C**) or cathepsin L (0.4 µg/mL) (**D**). Recordings began when cathepsin B in low pH buffer was pumped into the flow cell chamber (0 min) as well as at 5, 10, 15, 20, 25, and 30 min after. Residual Ca^2+^ was present from thermolysin treatment done to obtain cleaved GP. Error bars indicate standard error. (**E**) Unpaired two-tailed *t* test for the 30-min data point in the cumulative distribution functions is shown: *P < 0.05, ****P < 0.0001. All comparisons not shown are not statistically significant. (**F through H**) Cumulative distribution function for full fusion (**F**) and hemifusion (**G**) of EBOV GP_cl_ pseudovirus particles with SPEMs in the presence or absence of 100-µM E-64d. Recordings began when low pH buffer with or without E-64d was injected into the flow cell chamber (0 min) as well as 5, 10, 15, 20, 25, and 30 min after. Data points in panels C, D, F and G are averages obtained from four to six SPEMs under each condition. Error bars indicate standard error. (**H**) Unpaired two-tailed *t* test for the 30-min data point in the cumulative distribution functions is shown: **P < 0.01, ***P < 0.001. All comparisons not shown are not statistically significant.

We prepared HIV pseudoviruses with EBOV GP lacking its mucin-like domain (GPΔ). The pseudovirus membrane was labeled with 1-µM DiD during viral production (see Materials and Methods) so as to label both leaflets of the pseudovirus envelope. For most experiments, the glycan cap was proteolytically removed with thermolysin (19) to generate the 19-kDa form of GP (GP_cl_). The infectivity of these 19-kDa pseudoviruses was largely maintained in the presence of DiD in the membrane (Fig. S6). This labeling strategy allowed us to distinguish between hemifusion and full fusion events (Fig. 4A).

**Fig 5:**
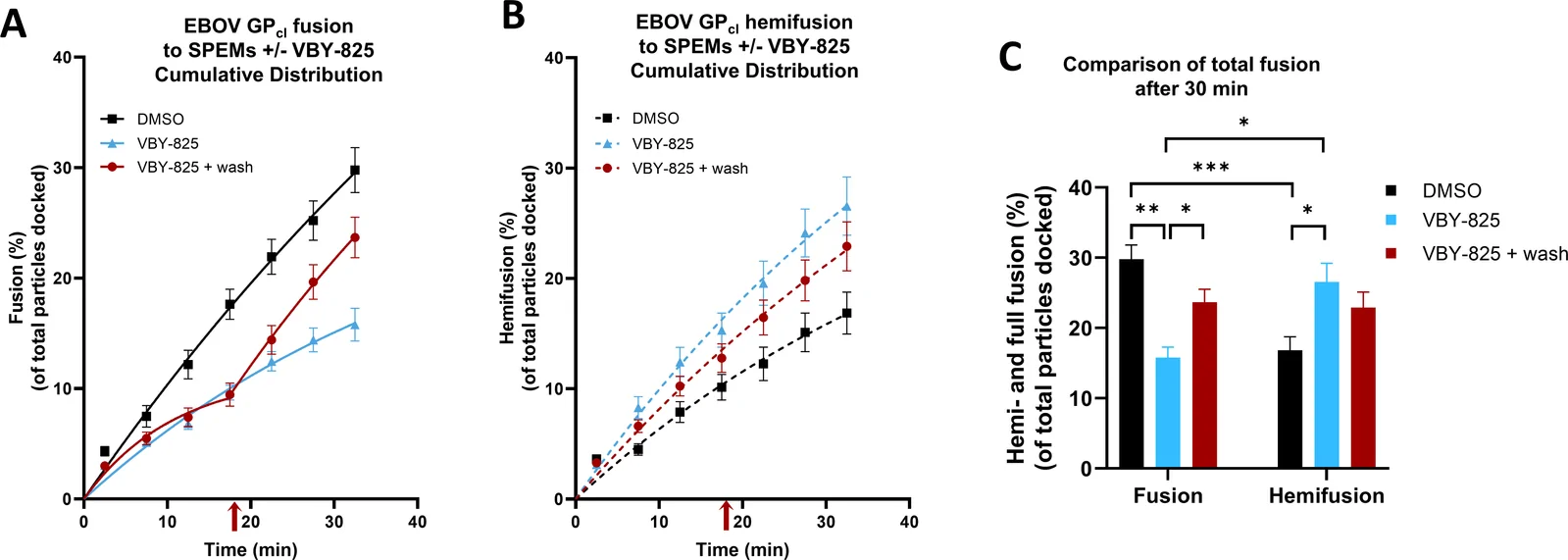
Effect of a reversible cathepsin inhibitor, VBY-825, on Ebola virus GP pseudovirus fusion to SPEMs. (**A through C**) Cumulative distribution functions for full fusion (**A**) and hemifusion (**B**) of EBOV GP_cl_ pseudovirus particles with SPEMs in the presence or absence of 1-µM VBY-825. Recording began when low pH buffer, with or without VBY-825, was injected into the flow cell chamber (0 min) as well as at 5, 10, 15, 20, 25, and 30 min after. To test for reversibility of the inhibition, 1-µM VBY-825/low pH was introduced into the flow cells, and movies were collected at times 0, 5, 10, and 15 min, after which VBY-825 was washed out with low pH buffer (red arrow), and additional movies were collected at the 20-, 25-, and 30-min timepoints. All buffers contained 0.1% dimethyl sulfoxide (DMSO). Data points in panels are averages obtained from three to six SPEMs under each condition. Error bars indicate standard error. (**C**) Unpaired two-tailed *t* test for the 30-min data point in the cumulative distribution functions is shown. *P < 0.05, **P < 0.01, ***P < 0.001. All comparisons not shown are not statistically significant.

We first explored the dependence of fusion on GPΔ cleavage to 19-kDa as well as on pH and Ca^2+^. Minimal fusion was observed with GPΔ under any condition tested (Fig. 4B). The lack of fusion with GPΔ was not due to insufficient viral particles bound to SPEMs, as similar numbers of bound particles were seen for GPΔ and GP_cl_ (143 ± 18 and 161 ± 18 particles per field of view for GPΔ and GP_cl_, respectively). GPΔ particles are presumably bound to attachment factors in the SPEMs (i.e., not to NPC1). The data further suggest that the membrane-associated cathepsin retained in the SPEMs (see next section and Fig. 5) is not sufficient to cleave GPΔ and/or that this membrane-associated cathepsin cannot easily access the GPΔ cleavage site. With GP_cl_, minimal fusion was observed at pH 7.4, but significant fusion was observed at pH 5.5, which was further enhanced by the addition of Ca^2+^ (0.5-mM CaCl_2_) (26) (Fig. 4B). Addition of Mg^2+^ (0.5-mM MgCl_2_) resulted in a similar extent of GP_cl_-mediated fusion compared to the EDTA condition. The observed effects of low pH and Ca^2+^ are in agreement with those of Munro and coworkers for lipid mixing (26), but here the results extend to full fusion. Small differences in the extent of thermolysin cleavage to 19-kDa GP (GP_cl_) did not influence the extent of full or hemifusion (Fig. S8A through C).

Since we and others previously observed that infection mediated by pseudovirions bearing EBOV GP_cl_ is inhibited by the general irreversible cysteine protease inhibitor E-64d (19, 36), we reasoned that addition of cathepsin might enhance fusion, and so we examined the effect of adding cathepsins to the system. We pre-bound EBOV GP_cl_ pseudoviruses to SPEMs at neutral pH, introduced activated cathepsin B or L (CatB or CatL) to the flow chambers at pH 5.5, acquired 2.5-min-long movies every 5 min for a total time period of 30 min (Fig. S7), and then calculated and plotted the cumulative fusion relative to the time of the pH drop. Addition of CatB modestly augmented the extent of full fusion over time (Fig. 4C and E), whereas the extent of fusion with CatL was not statistically different from samples with no added cathepsin (Fig. 4D and E). Neither cathepsin had a statistically significant effect on hemifusion (Fig. 4C through E). These subsequent cathepsin treatments of pseudovirus particles containing 19-kDa GP1 (GP_cl_) did not affect the amount or apparent size of GP1 (Fig. S8D).

Having observed considerable full fusion after simply lowering the pH of pseudovirus particles pre-bound to SPEMs, which was only modestly enhanced by the addition of catB, we asked if SPEMs harbor cathepsin activity. We first did this by including the irreversible cysteine protease inhibitor, E-64d, in the fusion assay. SPEMs, which are extensively washed before use (see Materials and Methods), were incubated with 100-µM E-64d followed by the introduction and binding of EBOV pseudoviral particles to the SPEMs at neutral pH in the presence of 100-µM E-64d. Low pH buffer supplemented with 100-µM E-64d was then introduced into the flow chamber, and 2.5-min-long movies were collected and analyzed (Fig. S9) as above. E-64d caused a marked decrease in EBOV GP_cl_-mediated full fusion (Fig. 4F and H), suggesting that the SPEMs retain cathepsin activity. However, hemifusion efficiency remained largely unaffected by the E-64d treatment (Fig. 4G and H). We next analyzed the SPEMs for the presence of cathepsins. As expected, enzyme activity measurements indicated that the starting endosomes contain cathepsin activity (Fig. S10A). Surprisingly, however, immunofluorescence analysis indicated that the extensively washed SPEMs contain catB (Fig. S10B). Lastly, we assessed the effects of a reversible cathepsin inhibitor, VBY-825 (46). SPEMs were incubated with 1-µM VBY-825 for 10 min followed by the introduction and binding of EBOV pseudoviral particles to the SPEMs at neutral pH in the presence of 1-µM VBY-825. Low pH buffer supplemented with 1-µM VBY-825 was then introduced into the flow chamber, and 2.5-min-long movies were collected as above (Fig. S11). VBY-825 resulted in reduced EBOV GP_cl_-mediated full fusion (Fig. 5A, blue vs black data points). Strikingly, fusion resumed immediately upon removal of VBY-825 (Fig. 5A, red data points and arrow), consistent with reversible inhibition of both cathepsin and fusion activities. Hemifusion was not altered upon washout of VBY-825 (Fig. 5A and C) and appeared to be enhanced by the DMSO vehicle (Fig. 5B and C; Fig. S12). Collectively, the data indicate that SPEMs retain membrane-associated cathepsin activity and that in addition to generating 19-kDa GP1 enabling binding to NPC1, cathepsins have an additional enhancing effect on the fusion of EBOV GP_cl_ pseudovirions with endosome membranes at low pH.

## DISCUSSION

In this study, we developed supported planar endosomal membranes (SPEMs) based on endosomes derived from HEK293T cells as a platform for studying virus entry through endosomes, as initially applied to enveloped virus fusion. Fusion assays employing SPEMs are capable of monitoring both hemifusion and full fusion. We first validated the system by demonstrating the low pH dependence of influenza virus and LASV fusion as well as the enhancement of low pH-dependent LASV GP pseudovirion fusion by its intracellular receptor, Lamp1 (by comparing fusion to SPEMs prepared from WT and Lamp1-null endosomes). We next explored fusion of pseudovirions bearing EBOV GP, for which full fusion has not been demonstrated with either model membranes or with the plasma membrane (22, 26, 31). For EBOV GP, we confirmed the need for its cleavage to the 19-kDa form and low pH as well as the enhancing effect of Ca^2+^ for lipid mixing (hemifusion) (26, 33), extending these observations to requirements for full fusion. Finally, we provided evidence (see below) that, as hypothesized (19), further cathepsin action enhances fusion mediated by 19-kDa EBOV GP.

SPEMs have many potential applications. In addition to value in assessing ionic requirements for hemifusion and full fusion (e.g., pH, Ca^2+^), SPEMs can also be used to examine the roles of endosome-specific lipids (e.g., bis(monoacylglycerol)phosphate (BMP)) or endosomal viral receptors. We observed a difference in the fusion profile of LASV GP-mediated fusion across a pH range from 4.5 to 7.4 in the presence and absence of the LASV intracellular receptor, Lamp1. EBOV and LASV were the first viruses shown to utilize intracellular receptors, NPC1 and Lamp1, respectively (20, 21, 44). Since those reports, other viruses have been shown to employ endosomal receptors: LCMV interacts with CD164 (47) and simian hemorrhagic fever virus and Lujo virus interact with CD63 (48, 49). It will be interesting to determine if these other endosomal receptors are absolutely required or alter the pH dependence of fusion, as we and others have seen for LASV (45, 50, 51). SPEMs may also have utility in studying the mechanisms of bilayer breaching by non-enveloped viruses and acid-activated bacterial toxins (52, 53).

EBOV entry into cells is complex. Studies over the past two decades have delineated major requirements including cleavage of EBOV GP1 to the 19 kDa form by the endosomal cysteine proteases CatB and CatL (19, 54, 55), binding to its endosomal receptor, NPC1 (20, 21), and fusion in late endosomes/lysosomes (36, 56), with low pH and Ca^2+^ inducing subtle conformational changes in the GP2 subunit (24, 26, 33). Conformational changes observed in the crystal structure of NPC1-bound GP_cl_ suggest that binding to NPC1 facilitates release of the fusion loop (57). Our early work indicated that in intact cells, low pH-dependent infection by pseudoviruses bearing 19 kDa GP1 requires a further E-64d-sensitive step (35). Attempts, however, to reconstitute EBOV GP_cl_-catalyzed fusion at the plasma membrane in forced fusion experiments in response to low pH (22, 31), or even low pH plus NPC1, Ca^2+^, K^+^, BMP, reducing agent, elevated temperature or cathepsins B plus L, have been unsuccessful (31). Our prior inability to induce full fusion at the plasma membrane with 19 kDa GP pseudoviruses even in the presence of added cathepsins, may reflect a recent finding of Melikyan and coworkers who showed differences in LASV GP-mediated fusion pore formation with endosomal vs plasma membranes (in intact cells) (51). This further supports the benefit of using near-native endosomal membranes, as exemplified by the SPEM system we have developed here, for *in vitro* studies of the fusion activity of viruses entering cells through endosomes.

Ebola GP is cleaved to GP1 and GP2 during biosynthesis by furin (58) and GP1 is additionally cleaved during viral entry by cathepsins B and L to a 19-kDa form (19, 54, 55). The infectivity of EBOV particles bearing 19-kDa GP is sensitive to a cysteine protease inhibitor (E-64d) and agents that raise endosomal pH (35, 36). This suggested that an additional low pH-dependent proteolytic step is required for efficient fusion. We provide evidence that cathepsins not only prime Ebola GP to its 19-kDa form but also exert an additional role beyond NPC1 binding. Interestingly, CatB appears more effective in this late activation step than CatL (Fig. 4C and D). Studies have shown EBOV infection to be sensitive to CatB, but not CatL, inhibitors in Vero E6 (55) and dendritic (59) cells, which may explain why we see an increase in fusion when CatB is added. Because we saw only a modest increase in fusion with the addition of CatB (Fig. 4C and E), as well as a higher fusion efficiency than expected without additional cathepsin, we evaluated fusion in the presence and absence of the cysteine protease inhibitor E-64d. There was a marked decrease in full fusion efficiency (Fig. 4F), while hemifusion remained the same (Fig. 4G). These findings are consistent with findings presented by Spence et al. (36), who observed EBOV GP_cl_-mediated lipid mixing (hemifusion) in cells treated with E-64d but no infection in the presence of E-64d, as originally seen by Schornberg et al. (19). Furthermore, a reversible cathepsin inhibitor (VBY-825) reversibly inhibited 19-kDa GP-mediated full fusion to SPEMs (Fig. 5A), with fusion restored almost instantaneously upon inhibitor washout. Collectively, these findings suggest two possibilities for optimal full fusion by 19-kDa EBOV GP: (i) cathepsin B further cleaves NPC1-bound 19-kDa GP, enabling the fusion loop (24, 60) to insert deeply into the endosome membrane and mediate a full fusion reaction, or (ii) cathepsin B acts on an endosomal membrane factor to promote optimal fusion pore formation. Of note, to the limit of resolution of SDS gels, we did not observe a change in the amount or size of 19-kDa GP1 following incubation of pseudovirus particles bearing 19-kDa GP (GP_cl_) with cathepsin B or L (Fig. S8D). Also intriguingly, cathepsin W has been shown to promote influenza virus fusion without a detectable effect on either of the influenza surface glycoproteins, hemagglutinin or neuraminidase (61). The fact that fusion in the presence of either E-64d or VBY-825 is not completely inhibited suggests that other (non-cysteine) proteases may also contribute to this final step of fusion, for example, cathepsin D, an aspartic endoprotease found in endosomes and lysosomes. Future experiments are needed to test these possibilities.

Our findings also indicate the presence of membrane-associated cathepsins in SPEMs, which are extensively washed before adding virus particles (Fig. 4F; Fig. S10). While most cathepsins in endosomes and lysosomes are in soluble form in the lumen of these organelles, there is evidence of a small membrane-bound population (62). Cathepsins are synthesized in the endoplasmic reticulum. The propeptide form of cathepsin is transported through the Golgi stack, where it binds to mannose-6-phosphate (M6P) receptors. The M6P receptor-bound procathepsins are then trafficked to endosomes/lysosomes, where the low pH typically results in the dissociation of the enzyme-receptor complex (reviewed in reference (63)). However, not all cathepsins may be discharged from M6P receptors. Also, some cathepsins are sorted to endosomes independent of M6P receptors, and those cathepsins may remain bound to alternate receptors. M6P receptor-independent membrane association has been reported in endosomes for cathepsins B and D in hepatic cells (62) and cathepsin D in macrophages (64). Finally, the low-density lipoprotein (LDL) receptor and the LDL receptor-related protein 1 (Lpr1) have been shown to transport cathepsins B and D to lysosomes in mouse embryonic fibroblasts (65).

Triggering of membrane fusion by initial binding to a receptor followed by proteolytic action has been reported for other viruses, notably coronaviruses, including severe acute respiratory syndrome coronavirus 1 and severe acute respiratory syndrome coronavirus 2 (SARS-CoV-2), as well as Middle East respiratory syndrome coronavirus. Coronaviruses require proteolytic activation of their spike proteins by cell surface or endosomal proteases for successful fusion and infection (66 – 68). Furin cleavage primes the spike protein of SARS-CoV-2 during viral biosynthesis to its subunits S1 and S2. Following receptor binding, transmembrane serine protease 2, which resides in the plasma membrane, or cathepsin L (in endosomes) cleaves the spike protein at a specific locus (the S2′ site), triggering fusion during viral entry (68 – 73). Hence, there are parallels between filoviral and certain coronaviral fusion in requiring both receptors as well as pre- and post-receptor-binding proteolytic steps. Additionally, mildly low pH (74) and calcium (75) have been reported to be required for efficient SARS-CoV-2 infection, indicating additional parallels between the fusion mechanisms for these two virus families.

In summary, the *in vitro* SPEM system we have developed provides a powerful tool with which to study the mechanism of fusion of endosome-entering viruses. SPEMs should also provide an excellent platform to study the role of other viral proteins in fusion, for example, the role of the Ebola matrix protein (VP40) disassembly in promoting full fusion and genome delivery (76, 77), as well as effects of viral glycoprotein mutations on fusion. For example, the A82V substitution in EBOV GP, which arose early during the 2013–2016 outbreak in western Africa, has been shown to increase infectivity (78, 79), but the molecular interactions and steps that cause this enhancement have not yet been determined. SPEMs could also be used to explore the mechanism by which non-enveloped viruses and certain toxins penetrate endosomes and thereby infect or intoxicate host cells (52, 53).

## MATERIALS AND METHODS

### Cells, plasmids, and reagents

HEK293T/17 cells (ATCC), Lamp1 KO HEK293T/17 cells (clone 2F6, described by Hulseberg et al. [45]) and Madin Darby Canine Kidney II (MDCK II) cells (originally from Dr. Ari Helenius) were maintained in high-glucose Dulbecco’s Minimum Essential Media (DMEM) (Gibco), supplemented with 10% fetal bovine serum (FBS) (Atlanta Biologicals), 2-mM L-glutamine (Gibco), 1-mM sodium pyruvate (Gibco), and 1% antibiotic-antimycotic (Gibco). HeLa-derived TZM-bl cells (NIH AIDS Reagent Program) were maintained in high-glucose DMEM (Gibco), supplemented with 10% FBS (Atlanta Biologicals) and 1% antibiotic-antimycotic (Gibco). All cells were maintained at 37°C and 5% CO_2_.

Plasmids for pseudovirus production, pFSW-Tat and psPAX2, were gifts from Dr. Jeremy Luban (University of Massachusetts Medical School). pHIV-Rev was a gift from Dr. Wen Yuan (University of Virginia). The surface protein plasmids, EBOV GP_Δ-mucin_ (VRC6002) of the Kikwit strain of EBOV GP and Lassa virus GP (LASV Josiah strain in pCMV), were originally from Dr. Gary Nabel and Dr. Gregory Melikyan (Emory University), respectively.

Antibodies against the NPC1 C-terminal tail (ab134113), Lamp1 C-terminal tail (ab24170), and EEA1 (ab2900) were from Abcam. The antibody for Lamp1 with the epitope in the luminal region (H4A3) was from Developmental Studies Hybridoma Bank. The anti-NPC1 C-loop antibody (sc-271335) was from Santa Cruz Biotechnology. The succinate dehydrogenase complex A antibody (2E3GC12FB2AE2) and sodium-potassium ATPase antibody (MA1-16731) were from ThermoFisher Scientific. The antibody against the Golgi SNARE protein, GS28, was a gift from Dr. David Castle at the University of Virginia. The anti-Rab5 antibody was a gift from Dr. Reinhard Jahn at the Max Planck Institute for Biophysical Chemistry. The calnexin antibody (ADI-SPA-860-D) was from Enzo Life Sciences. The monoclonal mouse-α-GP1 H3C8 antibody was a kind gift from Carolyn Wilson at the Food and Drug Administration.

### FPV preparation and membrane labeling

Recombinant influenza A/FPV/Rostock/1934(H7N1) attenuated with a monobasic cleavage site was created with a 12-plasmid system (80) and grown as previously described (81). Briefly, 293T cells in infection medium (DMEM supplemented with 0.2% wt/vol bovine serum albumin (BSA), 0.1% fetal calf serum, 2-mM glutamine, 1% antibiotic-antimycotic) were transfected with Lipofectamine 2000 and 1 µg of each of eight plasmids encoding each segment of the FPV genome, and four plasmids expressing the components of the FPV ribonucleoprotein complex. Four hours after transfection, the media were changed to infection medium containing 1-µg/mL TPCK-trypsin. Two days after transfection, the supernatant was harvested and cleared by pelleting 5,000 × *g* for 10 min. The cleared supernatant was added to 90% confluent MDCK II cells in infection media and allowed to enter for 1 h at 37°C after which the media were replaced with infection media containing 1-µg/mL TPCK-trypsin. Two days after infection, cytopathic effect was observed and the supernatant was harvested, cleared by centrifugation as above, aliquoted and frozen at −80°C. To avoid mutations from passage in culture, each subsequent preparation of FPV was performed by infecting MDCK II cells as described above with one of the original aliquots.

To prepare membrane-labeled virus, MDCK II cells grown to 80% density in T175 flasks were washed with 5-mL warmed phosphate-buffered saline (PBS). Seven milliliters of infection media (DMEM supplemented with 1-g BSA, 10% Supplemented Calf Serum (SCS), 1% antibiotic-antimycotic, and 1% L-glutamine) mixed with one aliquot of previously prepared FPV was added and incubated for 1 h at 37°C. The media was then aspirated and replaced with 15-mL infection media containing 0.625-µM DiD (1,1′-dioctadecyl-3,3,3′,3′-tetramethylindodicarbocyanine, 4-chlorobenzenesulfonate salt) (ThermoFisher Scientific) and 1 µL of 2-µg/mL TPCK-trypsin per 1 mL of infection media. After 48 h, the culture supernatant was collected and cleared by centrifuging at 5,000 × *g* for 15 min at 4°C. The virus was pelleted through a 25% sucrose-HME buffer (20-mM 4-(2-Hydroxyethyl)piperazine-1-ethanesulfonic acid, N-(2-Hydroxyethyl)piperazine-N′-(2-ethanesulfonic acid) (HEPES), 20-mM 2-(N-Morpholino)ethanesulfonic acid, 4-Morpholineethanesulfonic acid (MES), 130-mM NaCl, 1-mM EDTA, pH 7.4) cushion and resuspended in HME buffer without sucrose, aliquoted and stored at −80°C until use.

### HIV pseudovirus production

Virions pseudotyped with Ebola virus GP_Δmucin_ (EBOV GPΔ) and Lassa virus GP were produced in HEK293T/17 cells using Lipofectamine 2000 (Invitrogen). Cells grown to 60–80% confluency were transfected with 4.65-µg psPAX2 (Gag-Pol helper vector), 6.25-µg pFSW-Tat, 1-µg pHIV-Rev, and 1.5-µg of EBOV GP or LASV GP. Four to six hours post-transfection, the media were replaced with phenol red free DMEM supplemented with 10% FBS, 2-mM L-glutamine, and 1-mM sodium pyruvate. Pseudovirus-containing media were collected 48-h post-transfection and clarified by low-speed centrifugation. HIV pseudoviruses were pelleted through a 25% sucrose-HME cushion and resuspended in HME buffer without sucrose. The pseudovirus preparation was aliquoted and stored at −80°C. The concentration of HIV p24 was measured by enzyme linked immunosorbent assay (ELISA) (82).

Infection of TZM-bl cells by HIV pseudoviruses was performed as described by Sarzotti-Kelsoe et al. (83). Firefly luciferase activity was measured 2 days post-infection with BriteLite reagent (PerkinElmer Life Sciences) in a plate reader (GloMax Explorer, Promega). HIV pseudovirus preparations were diluted in Opti-MEM (Gibco) to the same concentration of p24.

### HIV pseudovirus membrane labeling

The outer leaflet of pseudovirus membranes was labeled with the fluorescent membrane label, Atto488- dimyristoylphosphatidylethanolamine (Millipore-Sigma) essentially as described (40). Briefly, Atto488-DMPE was dried on the bottom of a glass test tube to remove chloroform/methanol solvent and resuspended in HEPES buffer (20-mM HEPES, 150-mM NaCl, pH 7.4) to a concentration of 1.8 µg/mL. Using the p24 concentration of HIV pseudovirus, the virus was mixed with the dye suspension in a mass ratio of 4.4:1. The mixture was incubated at room temperature in the dark for 3 h under slow rotation. To remove free Atto488-DMPE, the mixture was diluted to 1 mL in HEPES buffer, and HIV pseudoviruses were pelleted by spinning at 21,000 × *g* for 1 h at 4°C. Atto488-DMPE-labeled HIV pseudoviruses were resuspended in HEPES buffer and stored in the dark at 4°C and used within 24 h.

DiD labeling of HIV pseudoviruses was done as described in the FPV preparation and membrane labeling section above, which labels both leaflets of the viral envelope.

### Cleavage of EBOV GPΔ to produce 19-kDa EBOV GP

HIV pseudoviruses bearing EBOV-GPΔ were cleaved to the 19 kDa form of GP with 0.5-mg/mL thermolysin freshly prepared from powder (Millipore-Sigma) in the presence of 2-mM CaCl_2_. Samples were incubated for 60 min at 37°C, and the reaction was terminated by adding 500-µM phosphoramidon (Millipore-Sigma). Fresh thermolysin powder was obtained when GPΔ cleavage to 19 kDa waned, as assessed by Western blotting. Representative blots showing cleavage to 19-kDa GP, along with corresponding fusion data, are shown in Fig. S8A and B.

### Late endosome enrichment protocol

Late endosome enriched samples were prepared from HEK293T cells. Cells (5- to 10-cm plates) were grown to about 90% confluency, then scraped into phosphate-buffered saline, and pelleted by centrifugation for 10 min at 1,000 × *g* at 4°C. The cell pellet was washed in 3-mL homogenization buffer (10-mM HEPES, 3-mM imidazole, 250-mM sucrose, pH 7.4). Following the wash, the cell pellet was resuspended in 3-mL homogenization buffer supplemented with 1× protease inhibitor cocktail (ThermoFisher Scientific) and passed through a ball-bearing homogenizer with a 0.6368-cm bore and 0.6335-cm diameter ball (gift of Dr. David Castle). The homogenate was centrifuged for 15 min at 4,000 × *g* at 4°C to pellet the nuclei. Sixty percent Optiprep (iodixanol) (Millipore-Sigma) was mixed with buffer (30-mM MOPS, 270-mM sucrose, 6-mM EDTA, pH 7.2) to obtain 7% and 14% Optiprep solutions for discontinuous gradients. The post-nuclear supernatant (PNS) was collected and mixed with an equal volume of 50% OptiPrep solution to obtain a final concentration of 25% Optiprep. This mixture was then transferred to a Beckmann SW 40 tube and overlayed carefully with 4 mL of 14% OptiPrep solution and 4 mL of 7% OptiPrep solution. This gradient was spun for 1.5 h at 35,000 rpm at 4°C. A white band at the interface between 7% OptiPrep and 14% Optiprep was collected. This fraction, which was enriched in early and late endosomes, was dialyzed in a cassette with 10,000-kDa molecular weight cutoff (ThermoFisher Scientific) against 4 L HMA buffer (10-mM HEPES, 10-mM MES, 10-mM sodium acetate, 100-mM sodium chloride, pH 7.4). The protein concentration of the dialyzed preparation of endosomes was determined using a bicinchoninic acid assay (BCA Assay) protein assay kit (Thermo Scientific). The preparation of enriched endosomes was stored at 4°C and used within 72 h.

### Preparation of supported planar endosomal membrane

Supported planar endosome membranes were prepared using the Langmuir-Blodgett/vesicle fusion technique (38, 84). Quartz slides were cleaned by immersing in Piranha solution (3:1 mixture of 95% sulfuric acid and 30% hydrogen peroxide) for at least 15 min and thoroughly rinsing in deionized water. The first leaflet of the SPEM was prepared by Langmuir-Blodgett transfer directly onto the quartz slide. A lipid mixture of 4:1 brain phosphatidylcholine and cholesterol (Avanti Polar Lipids) with 3 mol% of 1,2-dimyristoyl-*sn*-gycero-3-phosphatidylethanolamine-PEG3400-triethoxysilane (84) in a chloroform solution was applied onto a water surface in a Nima 611 Langmuir-Blodgett trough to reach an initial surface pressure of ~10 to 15 mN/m. The solvent was allowed to evaporate for 10–15 min, after which the monolayer was compressed at a rate of 10 cm^2^/min to reach a surface pressure of 32 mN/m. After equilibration for 5–10 min, a clean quartz slide was dipped into the trough at a speed of 68 mm/min and slowly withdrawn at 5 mm/min while maintaining constant surface pressure at 32 mN/m. The quartz slides with the monolayer were dried in a desiccator overnight. To form the outer leaflet of the SPEM, a quartz slide was assembled in a custom-built microscopy flow cell, and a suspension containing endosomes diluted to 0.5 mg/mL (as determined by BCA) in HMA buffer was injected into the flow cell and incubated for 1.0–1.5 hours at room temperature. SPEMs were washed with 10 volumes of HMA buffer to remove excess, endosomes and other non-attached material.

We also tested whether endosome preparations could be frozen and stored at −80°C for use at a later time but determined that SPEMs prepared from frozen and thawed endosome preparations were malformed (no smooth appearance) compared to SPEMs from freshly prepared endosomes and no longer supported fusion of LASV pseudovirions, although docking of these viruses was unchanged. However, SPEMs may be prepared from endosomes stored at 4°C for up to 2 weeks with only a small loss (~25%) of fusion permissiveness (Fig. S13).

### Total internal reflection fluorescence microscopy

To study binding and/or fusion to SPEMs, fluorescently labeled pseudoviruses were diluted in HMA buffer to obtain roughly 100–200 particles per field of view. The pseudoviruses were injected into the flow cell, and the fluorescence was monitored over time using prism-based TIRF microscopy. For pseudoviruses with the membrane label Atto488-DMPE, the sample was excited with a 488-nm laser (OBIS 488-nm LX, Coherent), and the emission light was filtered through a dichroic mirror (DC505, Semrock) and a band-pass filter (BP535/40, Chroma). For DiD-labeled FPV and pseudovirus, the sample was excited with a 640-nm laser (OBIS 640-nm LX, Coherent), and the emission light was filtered through a dichroic mirror (DC660, Semrock) and a long-pass filter (LP665, Semrock). Videos were recorded by an EMCCD (DV887ESC-BV, Andor Technology) in frame transfer mode with an exposure time of 0.1 s. Laser intensity, shutter, and camera were controlled by a custom program written in LabView (National Instruments).

For experiments with cathepsins B and L, recombinant human cathepsin B, CatB (R&D Systems), was activated by incubating in HMA buffer pH 5.0 at room temperature for 15 min. Recombinant human cathepsin L, CatL (R&D Systems), was activated by diluting it in HMA buffer pH 5.5 and incubating on ice for 15 min. Ebola pseudoviruses were pre-bound to SPEMs at neutral pH and incubated for 5–7 min. Unbound virus was washed away with 10 volumes of HMA buffer pH 7.4. The cathepsins were further diluted in HMA buffer pH 5.5 to 0.4 µg/mL and introduced into the flow cells. Videos of 2.5 min were collected immediately following cathepsin/low pH addition to the flow cell (this was marked as time 0) and 5, 10, 15, 20, 25, and 30 min after from different field of views. Cumulative distribution functions of fusion efficiencies were derived from the data collected at the aforementioned time points and by extrapolating the observed fusion rate in 2.5- to 5.0-min intervals.

For experiments with the cysteine protease inhibitor, E-64d (Sigma), a stock solution of E-64d, was prepared by dissolving it in water. The SPEMs were incubated with 100-µM E-64d in HMA buffer at pH 7.4 for 10 min. Ebola pseudoviruses were flowed in HMA buffer pH 7.4 with 100-µM E-64d and incubated for 5–7 min. Unbound virus was washed away with 10 volumes of HMA buffer pH 7.4 with 50-µM E-64d. HMA buffer pH 5.5 with 100-µM E-64d was introduced into the flow cells, and 2.5-min videos were collected immediately following the addition of E-64d/low pH (time 0) and 5, 10, 15, 20, 25, and 30 min after. Cumulative distribution functions were derived from the data collected as described above.

For experiments with the reversible cathepsin inhibitor, VBY-825 (AdooQ BioScience), a stock solution of VBY-825 was prepared by dissolving it in DMSO. The SPEMs were incubated with 1-µM VBY-825 in HMA buffer at pH 7.4 for 10 min. Ebola pseudoviruses were flowed in HMA buffer pH 7.4 with 1-µM VBY-825 and incubated for 5–7 min. Unbound virus was washed away with 10 volumes of HMA buffer pH 7.4 with 1-µM VBY-825. HMA buffer pH 5.5 with 1-µM VBY-825 was introduced into the flow cells, and 2.5-min videos were collected immediately following the addition of VBY-825/low pH (time 0) and 5, 10, 15, 20, 25, and 30 min after. To test for reversibility of the inhibition, 1-µM VBY-825/low pH was introduced into the flow cells, and movies collected at times 0, 5, 10, and 15 min, after which VBY-825 was washed out with low pH buffer, and additional movies were collected at the 20, 25, and 30 min timepoints. For experiments with VBY-825, all buffers contained 0.1% DMSO. Cumulative distribution functions were derived from the data collected as described above.

Intensities of single particles from regions of interest of 5 pixels × 5 pixels over time were extracted with a custom-built LabView program and classified as representing docking without fusion or docking with fusion based on the following criteria: a rapid increase in intensity followed by multiple frames of similar intensity without translation of the particle more than four pixels was classified as binding. If the intensity of the particle remained the same for the duration of the acquisition or slowly bleached over 15 s or more, this was considered docking without fusion. If the intensity of a docked particle decayed to background, which is characteristic for two-dimensional diffusion of fluorophores away from the site of fusion, the event was classified as binding with fusion. To distinguish fusion events from undocking events, a minimal decay time cutoff of 0.3 s was used to classify a single event as fusion.

### Antibody staining of SPEMs

SPEMs were prepared as described above. After excess, unfused endosomes were washed out of the flow cell, blocking buffer (15% FBS in PBS) was flowed in and incubated with the SPEM for 1 h at room temperature. The primary antibody was diluted in blocking buffer at the indicated dilutions and incubated with the SPEM for 1 h. Unbound primary antibody was washed with 10 volumes of PBS. Secondary antibody conjugated to Alexa fluor 488 or 555 was diluted in blocking buffer and incubated with the SPEM for another hour at room temperature. Excess unbound secondary antibody was washed with 20 volumes PBS. The SPEM was then imaged on a TIRF microscope.

### Fluorescence recovery after photobleaching

The lateral diffusion of lipids and integrity of the SPEMs were studied using fluorescence recovery after photobleaching. Carboxyfluorescein-PE (Avanti Polar Lipids) of 1 mol% was included in the monolayer prior to the preparation of the SPEM. Bilayers were bleached in a pattern of parallel stripes by a strong laser pulse (85), and the mean intensities were obtained from images before and after the bleach pulse was fit by the model:


$$F\left(t\right)=F_{\infty }+\left(F_{0}-F_{\infty }\right)e^{\left(-Da^{2}t\right)}$$

where *F*_0_ and *F*_∞_ are the initial and final fluorescence intensities after bleaching, respectively; *a* = 2π/*p*, *P* is the stripe period (12.7 µm), and *D* is the lateral diffusion coefficient. The mobile fraction (m.f.), which is a percentage of observed fluorescence recovery within the time frame of a FRAP experiment (60 s), is given by


$$m.f.=\frac{F_{\infty }-F_{0}}{F_{pre}-F_{0}}\times 200$$

where *F*_pre_ is the fluorescence intensity before photobleaching. At least 10 regions on four independently prepared SPEMs and bilayers were sampled to determine the reported average values.

## SUPPLEMENTAL MATERIAL

Supplemental Figures spectrum.01908-23-s0001.docx 13 supplemental figures.

ASM does not own the copyrights to Supplemental Material that may be linked to, or accessed through, an article. The authors have granted ASM a non-exclusive, world-wide license to publish the Supplemental Material files. Please contact the corresponding author directly for reuse.
