# Supplementary material for: A novel in vitro system of supported planar endosomal membranes (SPEMs) reveals an enhancing role for cathepsin B in the final stage of Ebola virus fusion and entry
Source: Microbiol Spectr. 2023 Sep 20;11(5):e01908-23. doi: 10.1128/spectrum.01908-23 (PMC10581071; doi:10.1128/spectrum.01908-23)
Supplement: Supplemental Figures — 13 supplemental figures. [file spectrum.01908-23-s0001.docx]

**Supplemental Material**

**A novel *in vitro* system of supported planar endosomal membranes (SPEMs) reveals an enhancing role for cathepsin B in the final stage of Ebola virus fusion and entry**

Laura Odongo, Betelihem H. Habtegebrael, Volker Kiessling, Judith M. White, Lukas K. Tamm

**Supplemental Figures**


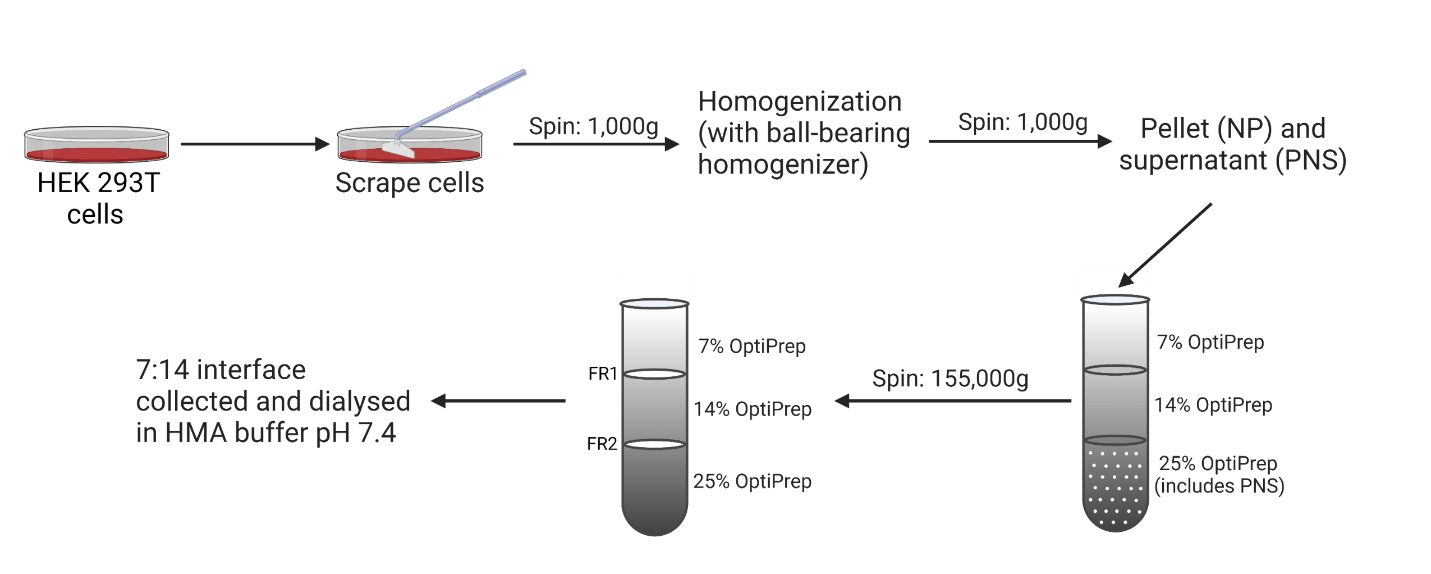


**Supplemental Figure 1. Endosome enrichment protocol.** Schematic diagram of subcellular fractionation using discontinuous Optiprep density gradient centrifugation. NP - nuclear pellet, PNS - post-nuclear supernatant, FR1 - Fraction 1: 7%/14% Optiprep interface, FR2 - Fraction 2: 14%/25% Optiprep interface.


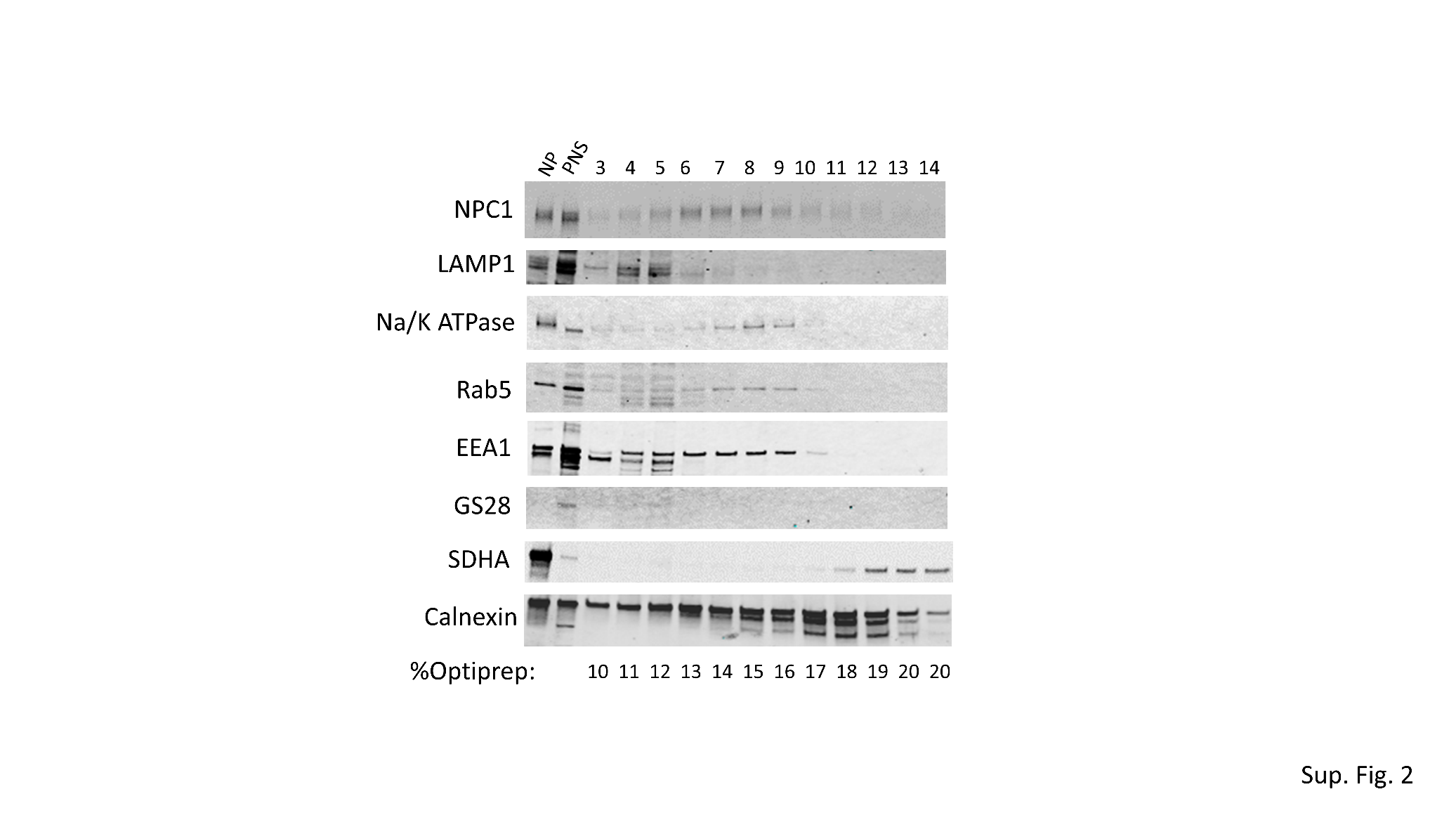
**Supplemental Figure 2.** Western blots of continuous gradient fractions probed for different organelle markers: late endosomes and lysosomes (NPC1 and Lamp1), early endosomes (EEA1 and Rab5), Golgi apparatus (GS28), mitochondria (SDHA – succinate dehydrogenase complex subunit A), ER (calnexin) and plasma membrane (Na/K ATPase). The organelles were separated on a continuous 5% to 20% Optiprep gradient. Equal volume fractions were collected from the top (fraction 3) to the bottom (fraction 14); the first two fractions were not analyzed. Analysis of fractions for organelle markers indicated that an enriched endosome fraction would band at a 7%/14% interface on a discontinuous Optiprep step gradient. The values below the blots are the Optiprep concentrations derived from the refractive indices of the respective fractions measured at room temperature. NP - nuclear pellet; PNS - post-nuclear supernatant.


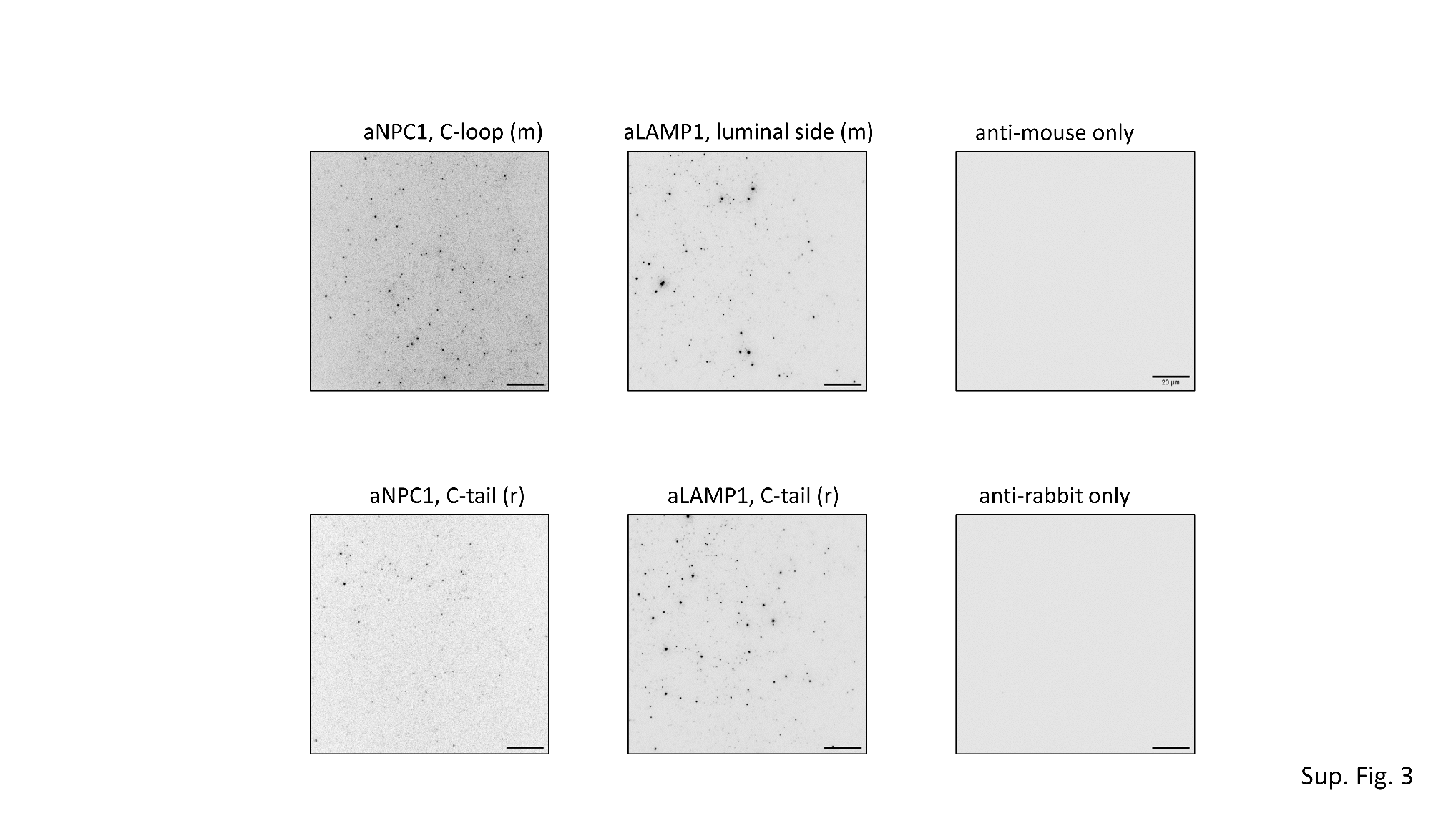
**Supplemental Figure 3. Assessment of sidedness of supported planar endosomal membranes (SPEMs).** Antibody staining of SPEMs showing orientation of endosomal membrane proteins. r - rabbit antibody, m - mouse antibody. The controls contain no primary antibody and only a donkey anti-mouse or donkey anti-rabbit antibody conjugated to Alexa fluor 555. All primary antibodies were used at a 1:100 dilution in blocking buffer (15% FBS in PBS). The signal-to-noise ratio was adjusted to make the signal more apparent, with the secondary antibody only controls being scaled similarly to the Lamp1 images. The images are shown in reverse contrast. Scale bar: 20 μm.


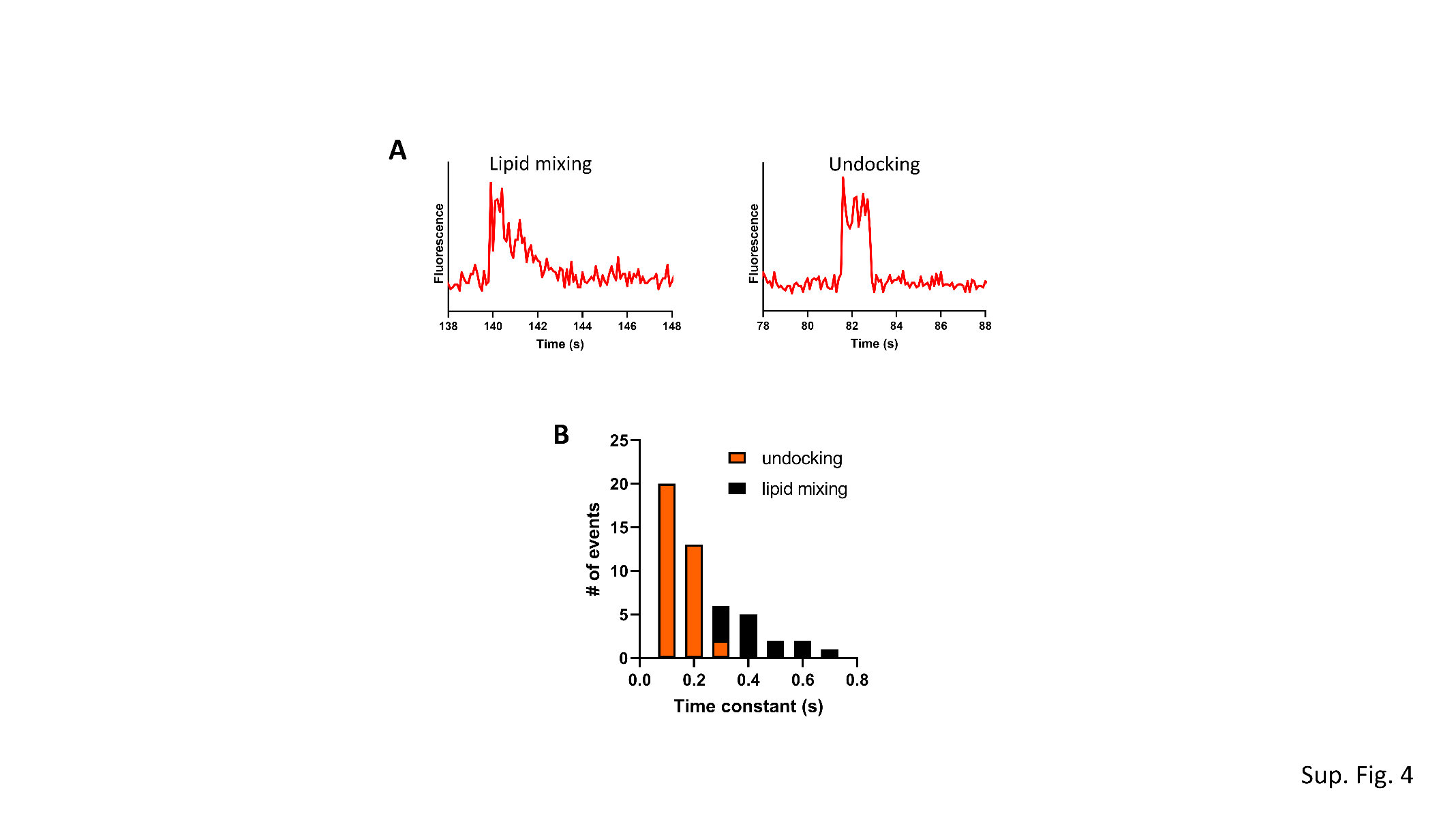


**Supplemental Figure 4. Decay time constants for lipid mixing and undocking events. A.** Fluorescence intensities of two different Lassa GP pseudovirus particles over time, with one undergoing lipid mixing (left) and the other undocking from the SPEM (right). **B.** Time constants of less than 0.3 s were denoted as undocking and those at or above 0.3 s were denoted as lipid mixing events. The lipid mixing and undocking events were taken from events observed in the SPEM with no Lamp1, pH 4.5 condition (Figure 3).


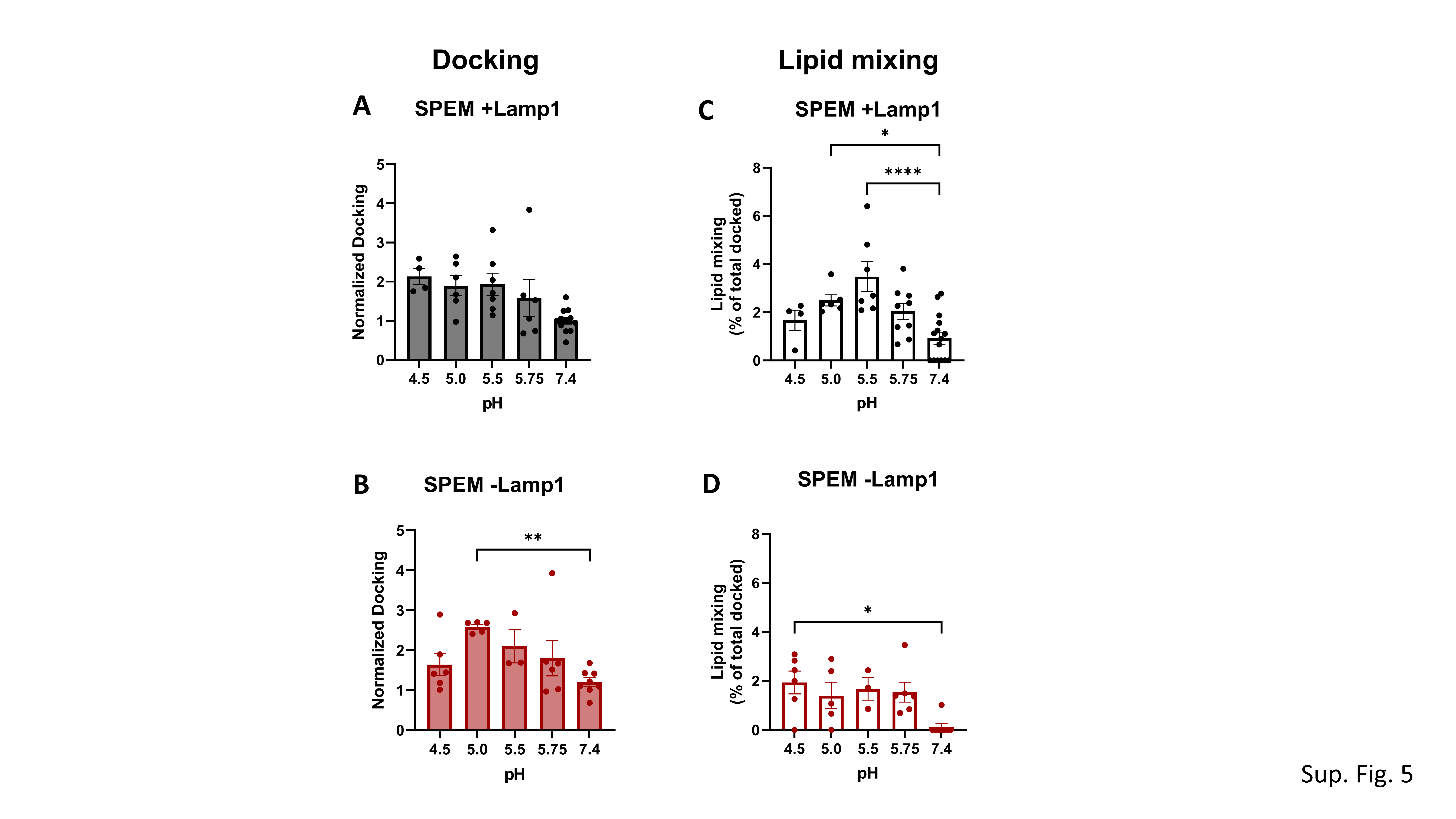
**Supplemental Figure 5. Docking and lipid mixing of Lassa GP pseudoviruses to SPEMs.** HIV pseudoviruses bearing Lassa GP and labeled with Atto488-DMPE (outer leaflet only) were flowed into the flow chambers with SPEMs and attached to and allowed to undergo lipid mixing with the SPEMs at the indicated pHs. **A and B.** Lassa pseudovirus associated with SPEMs with and without Lamp1 at the indicated pH. Docking for both SPEMs with and without Lamp1 was normalized to docking observed in the SPEM sample with Lamp1 at pH 7.4. **C and D.** Lipid mixing of Lassa GP pseudoviruses with SPEMs with and without Lamp1 at the indicated pH within 2.5 minutes. Each data point represents the average percent lipid mixing of approximately 75 to 220 docked particles observed on separately prepared SPEMs. These data from separately prepared SPEMs were further averaged and plotted in Fig. 3C. Error bars indicate standard error. One-way ANOVA test is shown above the data: *, *p* < 0.05; **, *p* < 0.01; ***, *p* < 0.001; ****, *p* < 0.0001. All comparisons not shown are not statistically significant.

**
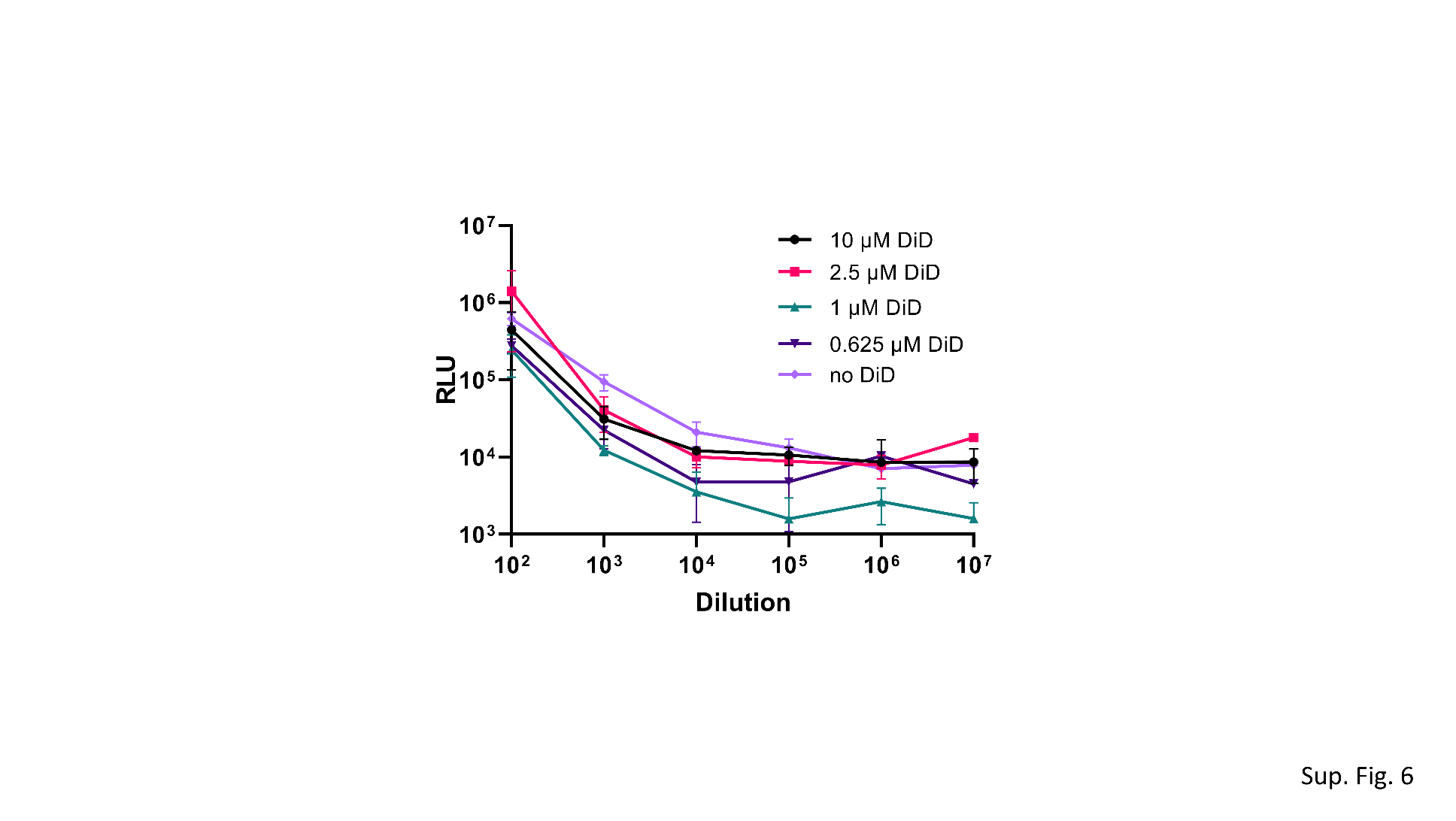
**

**Supplemental Figure 6. Infectivity of Ebola GP pseudoviruses labeled with different concentrations of DiD during viral production.** TZM-bl cells were incubated with serial dilutions of HIV particles pseudotyped with Ebola GPΔ that had been labelled with 10, 2.5, 1, 0.625 μM or no DiD (mock) in triplicate in 96-well culture plates. The luciferase activity was measured 48 hours after incubation and is expressed as RLU after subtraction of background luminesce from control wells. Triplicate wells, in a 96 well plate, were analyzed for each sample at each dilution. The infectivity was measured for two independent pseudovirus preps. The error bars indicate standard error calculated from two average values (from 3 replicates) for each preparation.


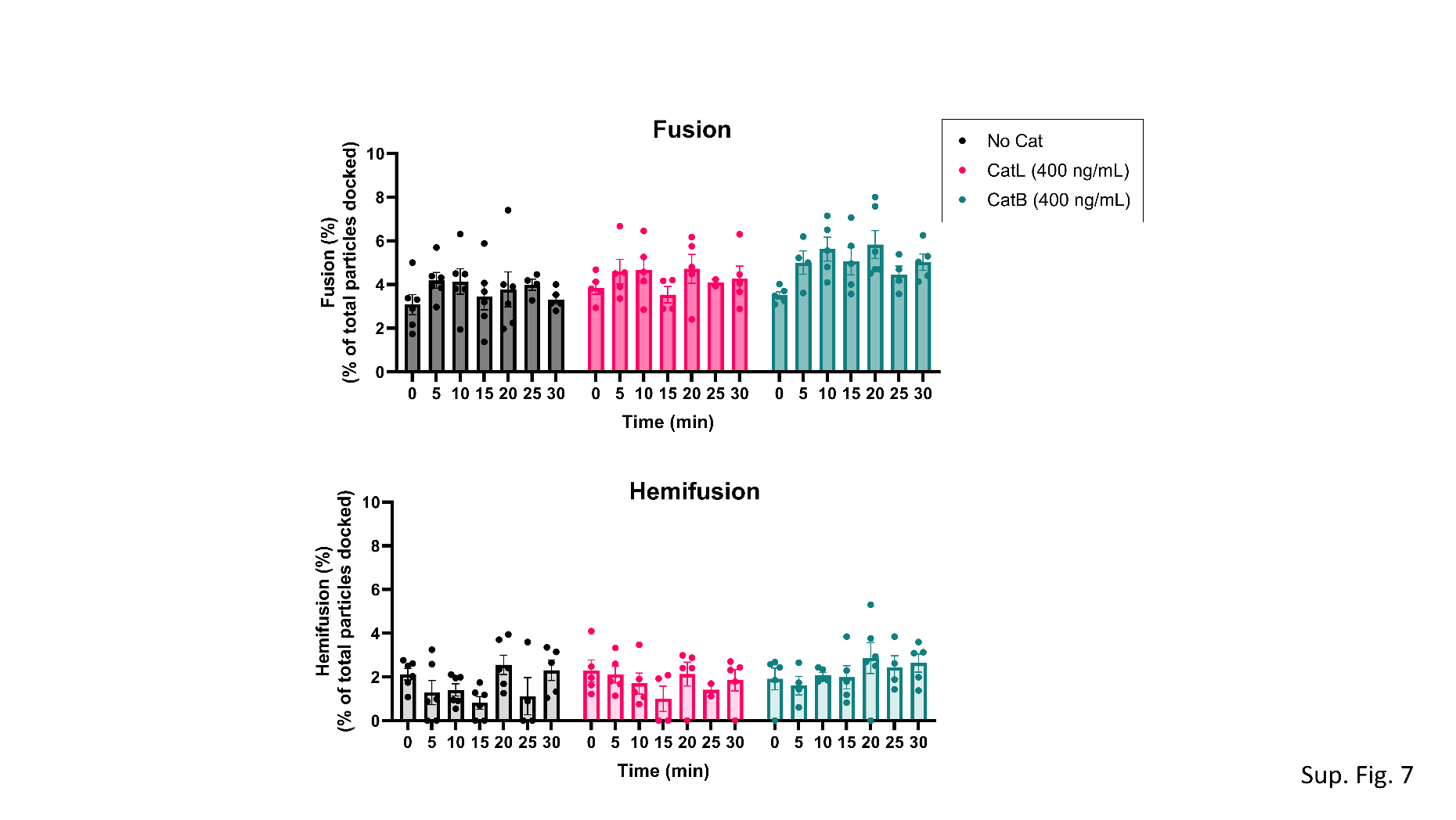
**Supplemental Figure 7. Fusion of Ebola pseudovirus with SPEMs in the presence or absence of cathepsin B or L at the indicated concentrations.** Movies were recorded immediately after 0.4 μg/mL cathepsin B or L (or no cathepsin) in low pH buffer was flowed into the flow cell chamber (0’) as well as at 5, 10, 15, 20, 25, and 30 minutes after. Residual Ca^2+^ was present from the thermolysin treatment done to obtain GP_cl_. Each data point represents events observed on one separately prepared SPEM. Error bars indicate standard error. The cumulative distribution functions of these data as well as statistical analyses are plotted in Figs. 4C, D and E.


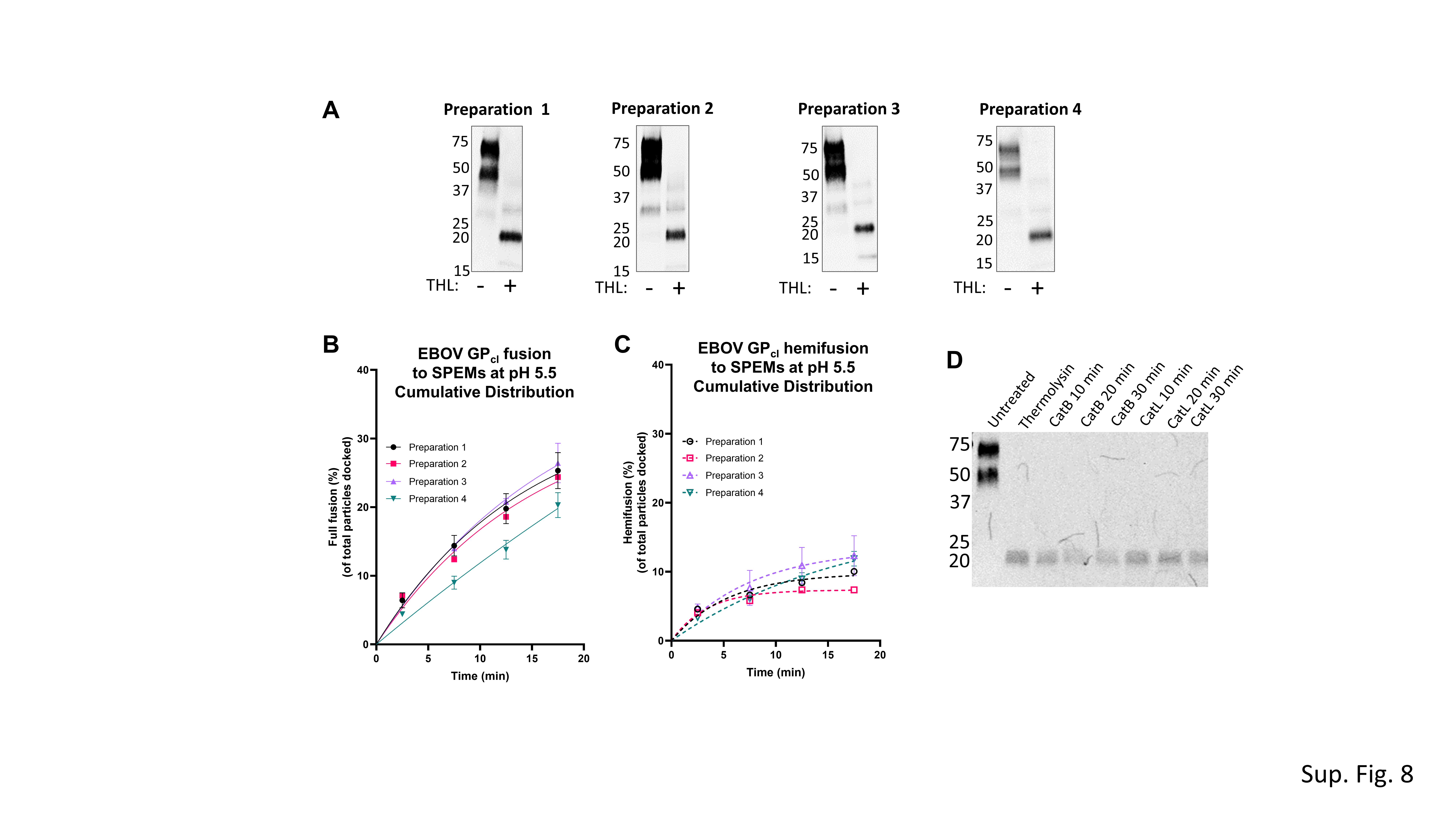
**Supplemental Figure 8**. **Fusion activity of** **pseudovirions containing** **thermolysin-cleaved Ebola virus GP (GPcl) and effects of subsequent treatment with cathepsin B or L on the migration of GP_cl_. A.** Western blots probed for GP1 of SDS gels of four preparations of DiD-labeled HIV particles pseudotyped with Ebola GPΔ and treated with thermolysin (THL) to generate 19-kDa GP (GP_cl_); preparations 1, 2 and 3 were used in Figs. 4C, D and preparation 4 was used in Figs. 4F, G. **B.** Cumulative distribution functions for full fusion **(B)** and hemifusion **(C)** of EBOV GP_cl_ pseudovirus particles with SPEMs; the data are the no cathepsin added controls from Figs. 4C,D and F,G. **D.** EBOV GPΔ was treated with thermolysin to generate GP_cl_ and then treated with 5 μg/ml CatB or CatL for 10, 20, or 30 minutes at 37°C. SDS gel samples were probed on western blots for GP1.


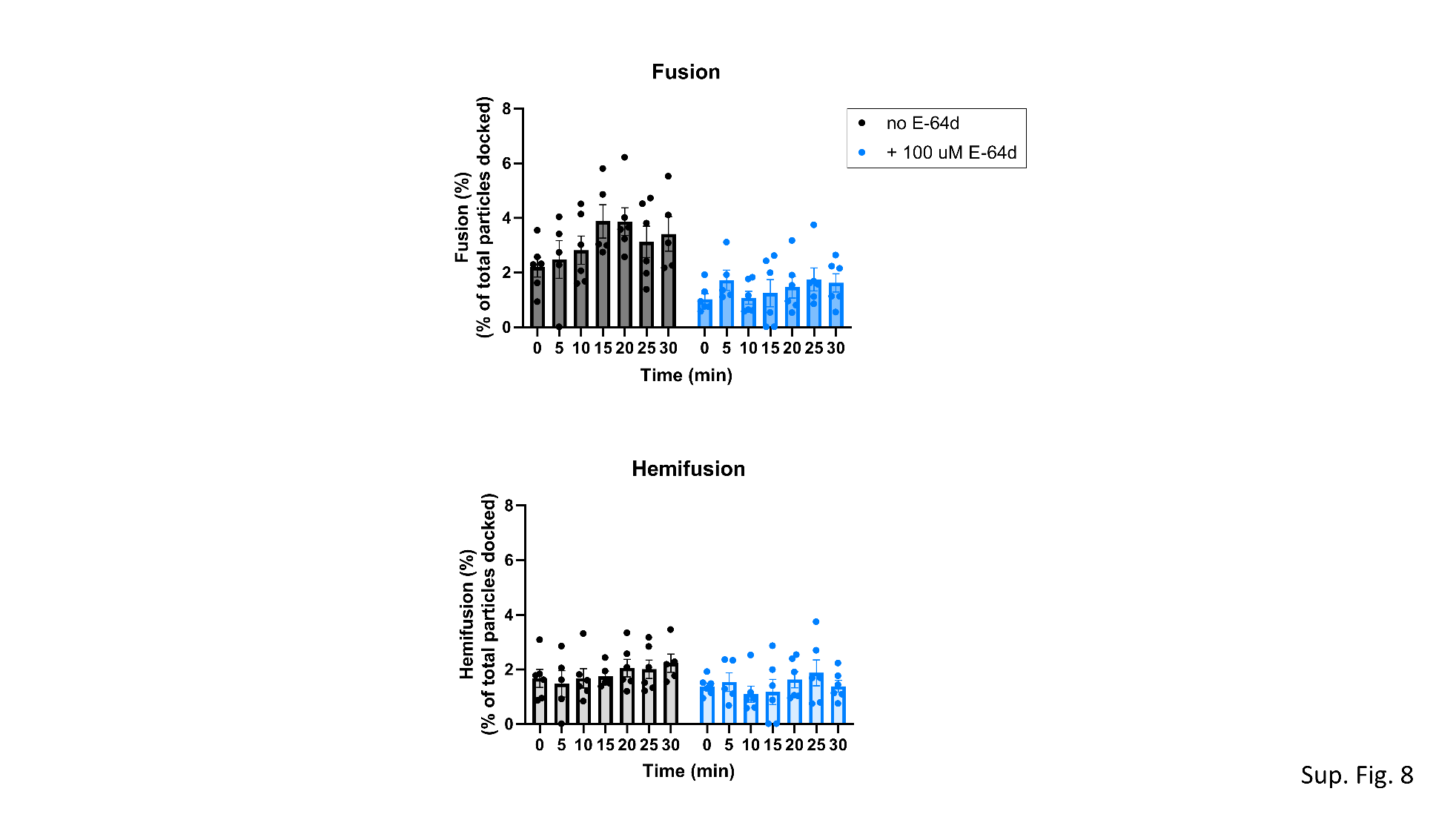
**Supplemental Figure 9. Fusion of Ebola pseudovirus with SPEMs in the presence or absence of the cysteine protease inhibitor, E-64d.** Movies were recorded immediately after 100 μM E-64d in low pH buffer or only low pH buffer was flowed into the flow cell chamber (0’) as well as at 5, 10, 15, 20, 25, and 30 minutes after. Residual Ca^2+^ was present from the thermolysin treatment done to obtain GP_cl_. Each data point represents events observed on one separately prepared SPEM. Error bars indicate standard error. The cumulative distribution functions of these data as well as statistical analyses are plotted in Figs. 4F, G and H.


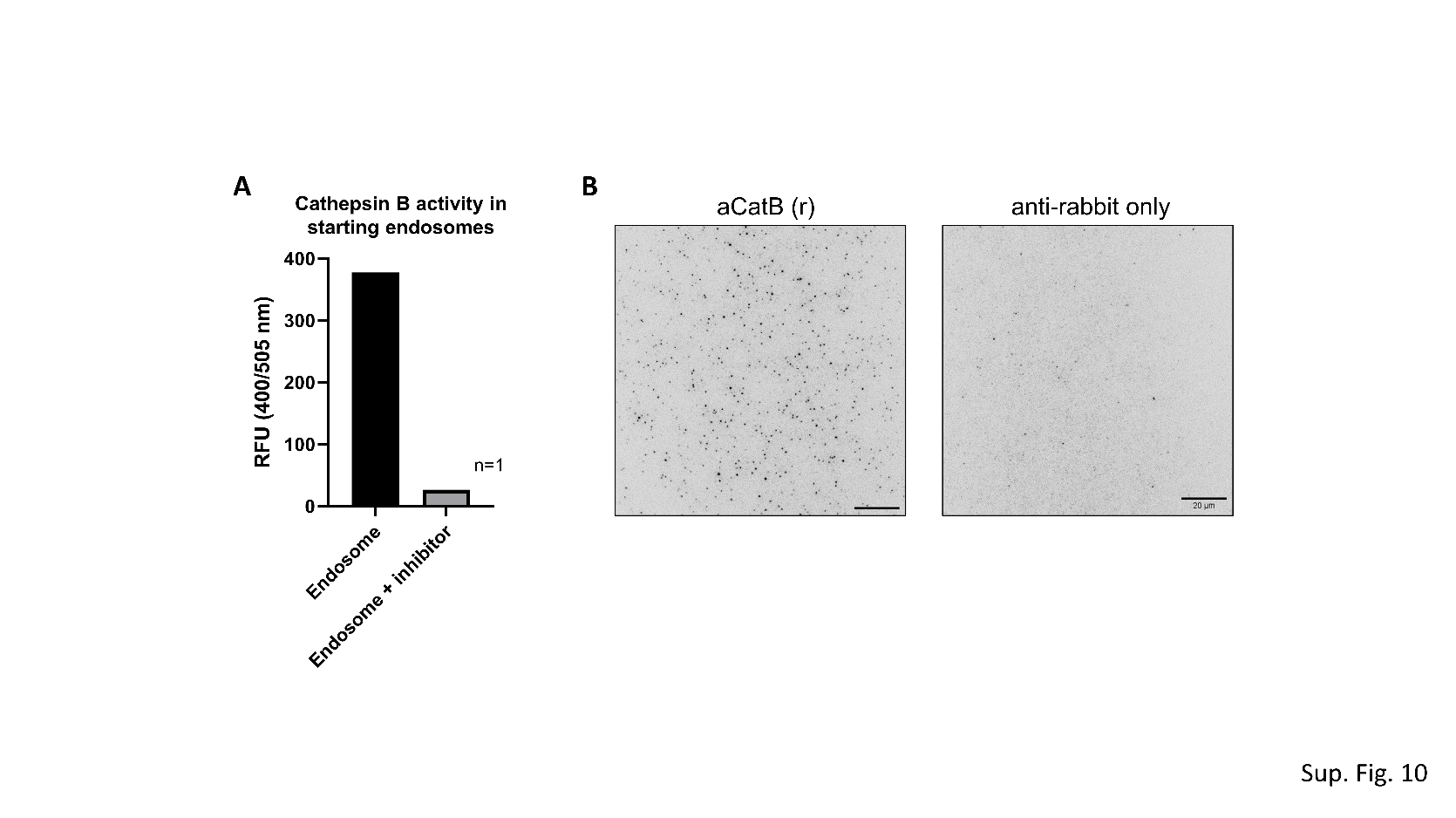
**Supplemental Figure 10. Assessment of the presence of cathepsin B in washed SPEMs. A.** Cathepsin B activity in endosomes derived from HEK 293T cells with or without the addition of inhibitor. **B.** Antibody staining of SPEMs showing the presence of cathepsin B. r – rabbit antibody. The controls contain no primary antibody and only a donkey anti-rabbit antibody conjugated to Alexa fluor 488.The cathepsin B antibody was used at a 1:2000 dilution in blocking buffer (15% FBS in PBS). The signal-to-noise ratio was adjusted to make the signal more apparent, with the secondary antibody only control being scaled similarly to the CatB image. The images are shown in reverse contrast. Scale bar: 20 μm.


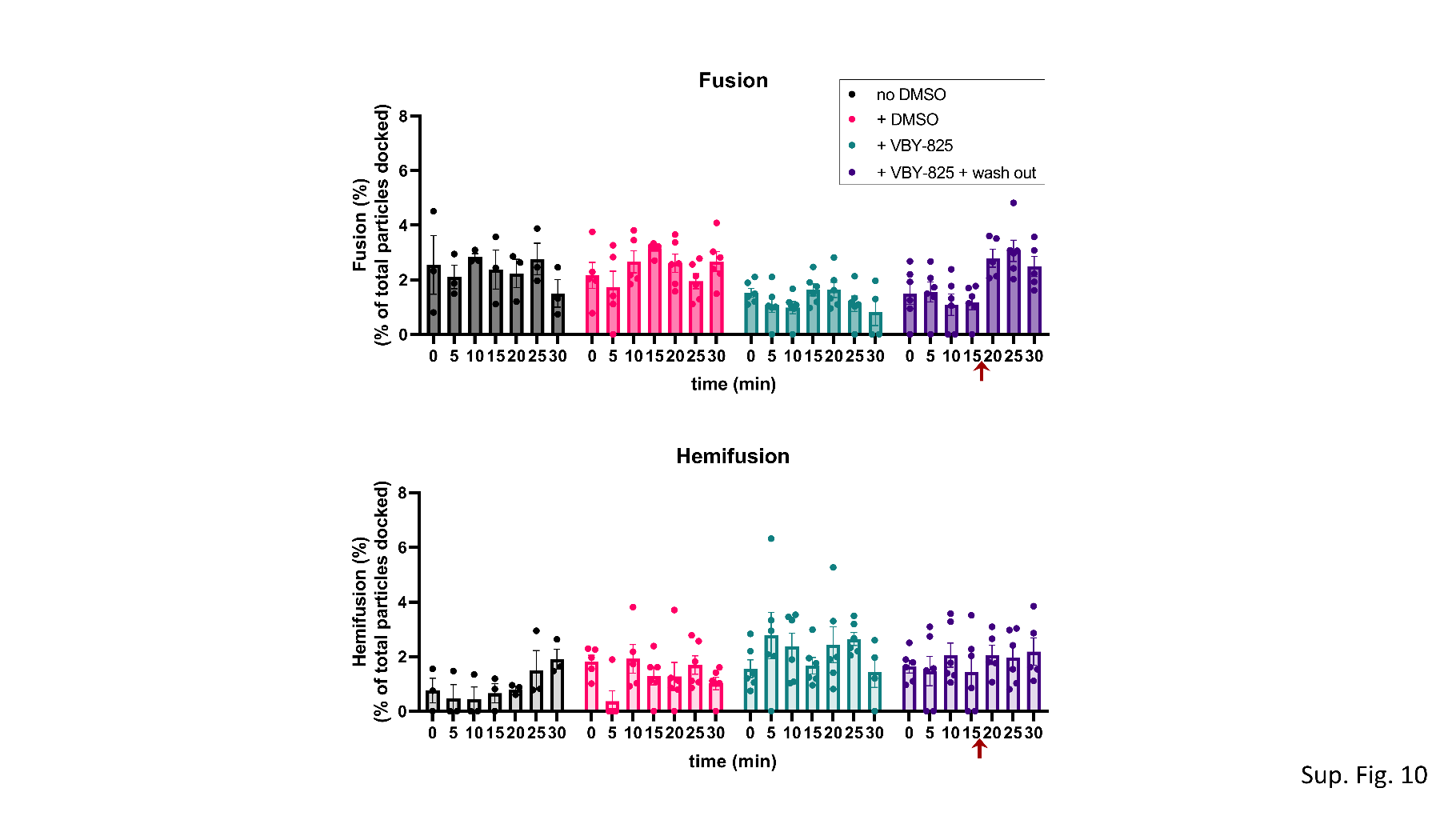
**Supplemental Figure 11. Fusion of Ebola pseudovirus with SPEMs in the presence or absence of the reversible cathepsin inhibitor, VBY-825.** Movies were recorded immediately after 1 μM VBY-825 in low pH buffer or DMSO (in lieu of VBY-825) in low pH buffer was flowed into the flow cell chamber (0’) as well as at 5, 10, 15, 20, 25, and 30 minutes after. To test for reversibility of the inhibition, 1 μM VBY-825/low pH was introduced into the flow cells and movies collected at times 0, 5, 10 and 15 min, after which time VBY-825 was washed out with low pH buffer (red arrows) and additional movies were collected at the 20, 25, and 30 min timepoints. All buffers, except the ‘no DMSO’ condition, contained 0.1% DMSO. Residual Ca^2+^ was present from the thermolysin treatment done to obtain GP_cl_. Each data point represents events observed on one separately prepared SPEM. Error bars indicate standard error. The cumulative distribution functions as well as statistical analyses of these data are plotted in Fig. 5.

**
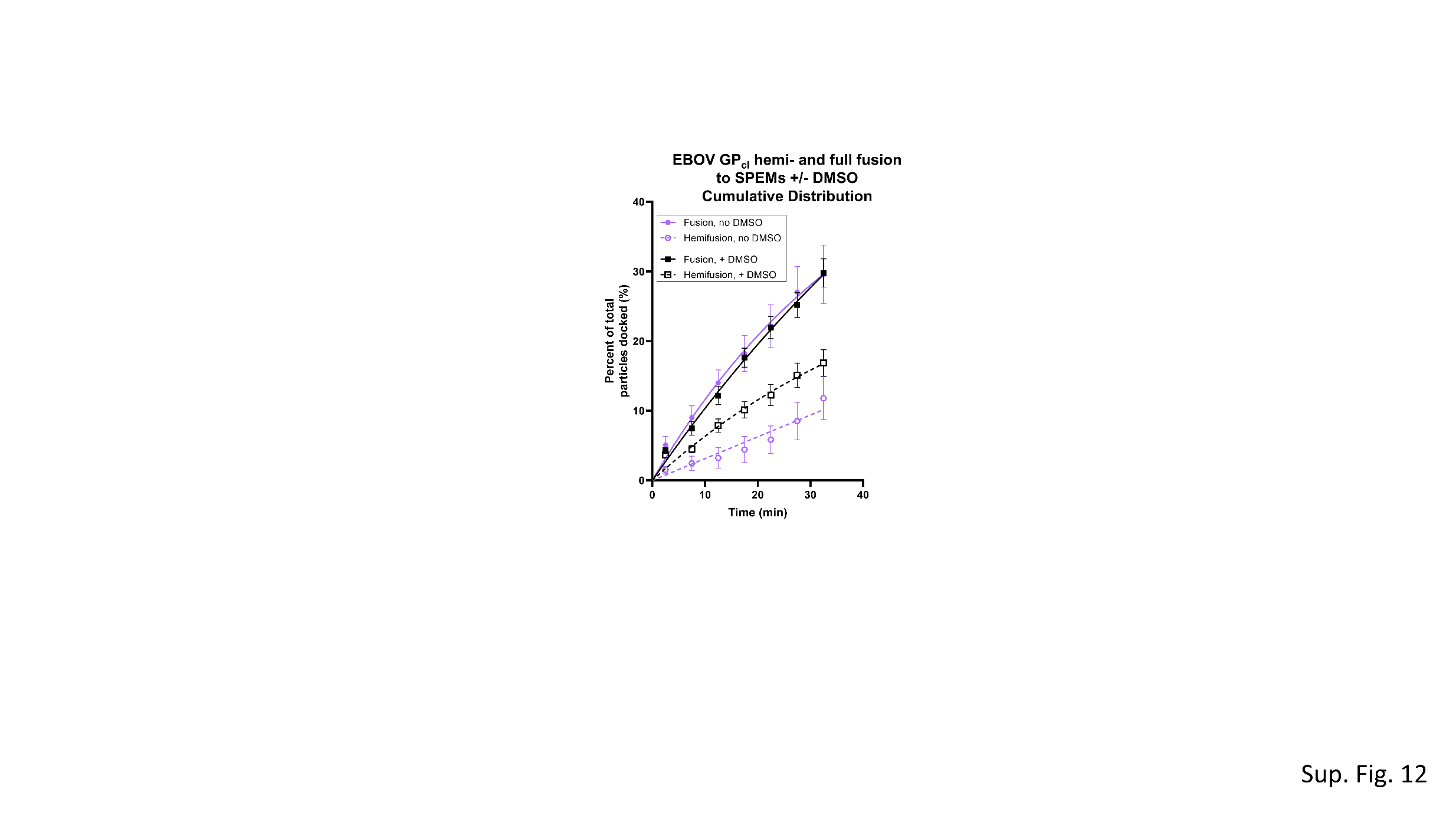
Supplemental Figure 12. Effect of DMSO on Ebola virus GP_cl_ pseudovirus fusion to SPEMs.** Cumulative distribution function for full fusion and hemifusion of EBOV GP_cl_ pseudovirus particles with SPEMs in the presence or absence of 0.1 % DMSO. Movies were recorded immediately after low pH buffer, with or without DMSO, was injected into the flow cell chamber (0’) as well as at 5, 10, 15, 20, 25 and 30 minutes after. Residual Ca^2+^ was present from the thermolysin treatment done to obtain GP_cl_. Error bars indicate standard error. These data were acquired as controls for Fig. 5.


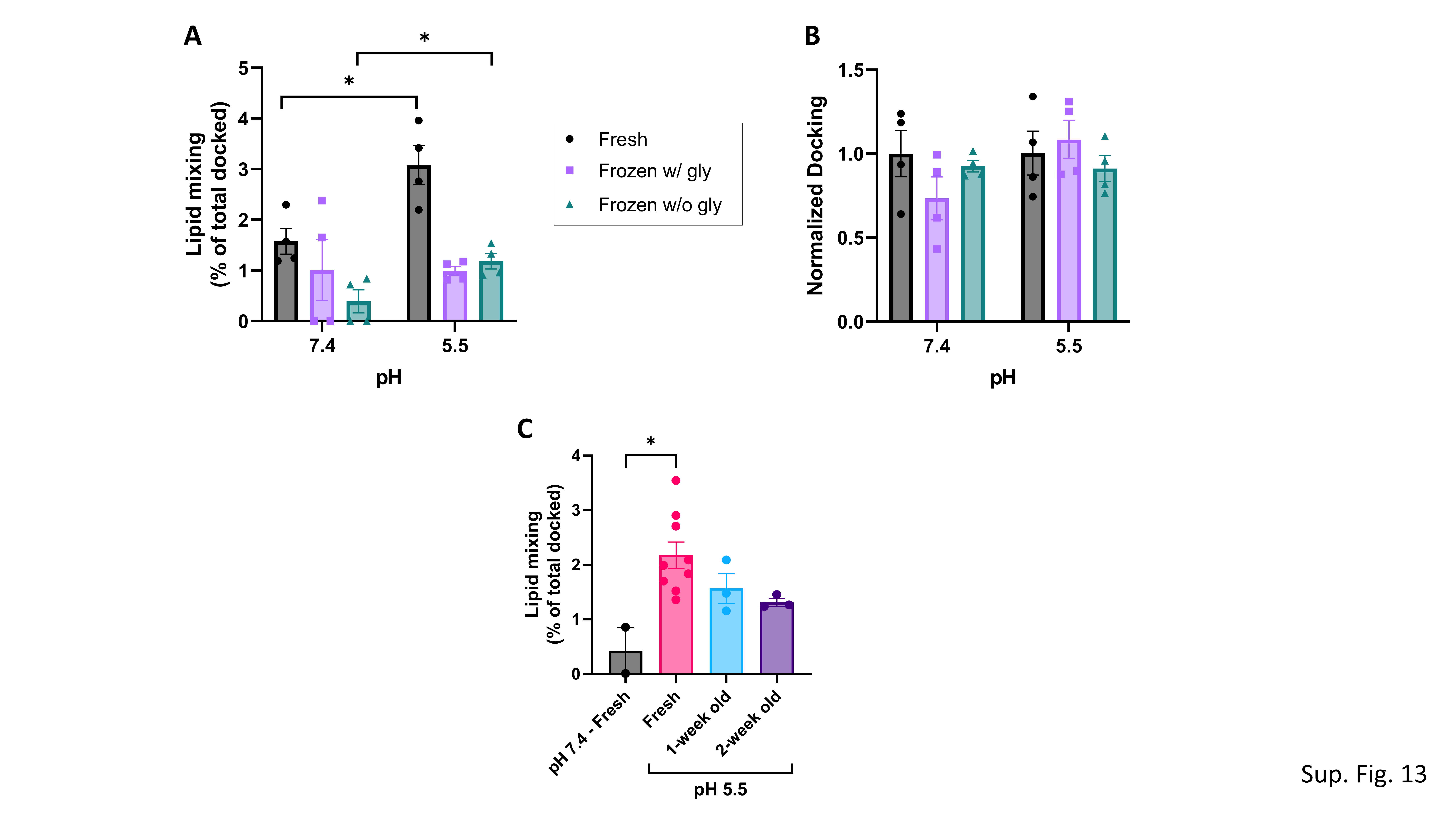
**Supplemental Figure 13. Assessment of LASV GP-mediated fusion to SPEMs prepared from endosomes stored under different conditions.** HIV pseudoviruses bearing Lassa GP and labeled with Atto488-DMPE (outer leaflet only) were flowed into the flow chambers with SPEMs and allowed to attach to and undergo lipid mixing with the SPEMs at pH 7.4 and 5.5. **A.** Lipid mixing of Lassa pseudovirus particles with SPEMs prepared from freshly extracted endosomes, endosomes supplemented with 10% glycerol (gly) and stored at ‑80°C (w/ gly) and endosomes without gly stored at -80°C (w/o gly) for 2-3 weeks. **B.** Docking of Lassa pseudovirus to SPEMs prepared from freshly extracted endosomes, endosomes supplemented with 10% glycerol and stored at -80°C (w/ gly) and endosomes without gly stored at -80°C (w/o gly) for 2-3 weeks. **C.** Lipid mixing of Lassa pseudovirus with SPEMs prepared from freshly extracted endosomes, and endosomes stored at 4°C for one or two weeks. For A-C, error bars indicate standard error. Statistical analysis was performed using unpaired two-tailed *t* test: *, *p* < 0.05. All comparisons not shown are not statistically significant.
